# Expanding the Coordination Chemistry of Decavanadate through π‑Hole Interactions with Transition-Metal Cyclen Complexes: Electronic Features and Dye Adsorption

**DOI:** 10.1021/acs.inorgchem.6c00422

**Published:** 2026-03-11

**Authors:** Heloísa de Souza Camilo, Lucas Gian Fachini, Lorena Moreira Braga, Gabriel Barros Baptistella, Juliana Morais Missina, Grazielli da Rocha, Francine Bertella, Patrizia Rossi, Paola Paoli, Eduardo Lemos de Sá, Giovana Gioppo Nunes

**Affiliations:** † Departamento de Química, 355751 Universidade Federal Do Paraná, Curitiba, Paraná 81530-000, Brazil; ‡ Dipartimento di Ingegneria Industriale, 9300 Università Degli Studi di Firenze, Firenze 50139 Italy

## Abstract

The hybrid decavanadate with macrocycle cyclen (1,4,7,10-tetraazacyclododecane) complexes with the formula [Ni­(cyclen)­(H_2_O)_2_]_2_[H_2_V_10_O_28_]·2H_2_O (**1**) was prepared as an ionic pair, while [{Cu­(cyclen)}_2_(H_2_V_10_O_28_)]·9H_2_O (**2**) and [{Zn­(cyclen)}_3_(V_10_O_28_)]·4H_2_O (**3**) were obtained as discrete molecular entities. The structures of **2** and **3** revealed a new coordination mode for the decavanadate anion, involving a triply bridging oxygen (−μ_3_-O_B_) and {TM­(cyclen)}^2+^, where TM = Cu­(II) or Zn­(II). Electrostatic Surface Potential analysis showed pronounced π-holes in the {Cu­(cyclen)}^2+^ and {Zn­(cyclen)}^2+^ fragments, whereas {Ni­(cyclen)}^2+^ lacks this feature due to stronger metal-cyclen σ-bonding. The Independent Gradient Model based on Hirshfeld partition analysis of {TM­(cyclen)}/decavanadate interfaces demonstrated that intramolecular noncovalent interactions play a key role in structural stability. The low Intrinsic Bond Strength Index for the Cu–O_B_ bond (0.087) suggests a weak, semicoordinative interaction with approximately half the strength of the Zn–O_B_ coordination bond (0.169). The adsorption of methylene blue was characterized as a surface phenomenon. The bleaching efficiency, **2** > **1** > **3**, was determined by the distribution of their asymmetric surface charge and particle size. These hybrid compounds provide a valuable model for understanding and advancing decavanadate coordination chemistry and for the application of polyoxometalates to remove prevalent contaminants from wastewater.

## Graphical abstract

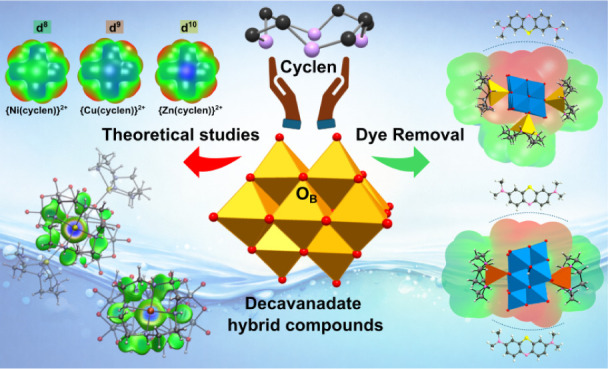

## Introduction

Polyoxometalates (POMs) are regarded as one of the most versatile classes of inorganic materials due to their diverse compositions, intriguing architectures, and physical and chemical properties.^1^ Furthermore, one or more transition metal (TM) complexes may be combined with POMs, either as an ion pair to provide charge compensation or as part of the inorganic framework itself. The latter can lead to high-dimensional frameworks, commonly based on Lindqvist-type structures, or large Keggin-type and Wells-Dawson-type polyoxidoanions.^2^ Such hybrid POM–metalorganic architectures have found applications in the fields of biomedicine,^3^ catalysis,^4^ adsorption^5^ and electronic devices.^6^ The combination of discrete POMs with cationic metal complexes of organic macrocycles creates unique systems that incorporate two building blocks with distinct structural characteristics.^7–10^ Among those, Co­(III), Ni­(II), Zn­(II) and Cu­(II) azamacrocycle complexes have been assembled with Lindqvist-type [M_6_O_19_]^2–^ isopolyanions (M = Mo­(VI), Nb­(V) and W­(VI)), where the latter represent a unique case of spontaneous optical resolution of a racemic complex assisted by a POM.^11–13^

The decavanadate anion [H_x_V_10_O_28_]^(6–x)–^ (x = 0 to 4) (V_10_, Figure 1) is the most studied polyoxovanadate (POV), mainly due to its relatively simple synthesis, stability in acidic media (pH 3–6), biological relevance,^14^ occurrence as natural minerals,^15^ and interesting photo- and redox-properties.^16^ Regarding TM azamacrocycle complexes combined with V_10_, one of the few examples includes an open framework with a {Cu­(cyclam)}^2+^ fragment, where cyclam = 1,4,8,11-tetraazacyclotetradecane.^9^ This extended structure exhibited permanent microporosity, selectivity for the adsorption of CO_2_, and catalytic oxidation activity toward tricyclic alkanes. Cyclen (1,4,7,10-tetraazacyclododecane), in turn, is another macrocycle widely employed in supramolecular chemistry that remains unexplored in assemblies containing V_10_. The nitrogen atoms of cyclen have a high affinity for TM ions, promoting strong metal complexation, associated with their high thermodynamic stability and relative kinetic inertness.^17^ Due to the structural differences between cyclam and cyclen complexes, topological diversity could be expected for supramolecular materials based on these macrocycles.

**Figure 1:**
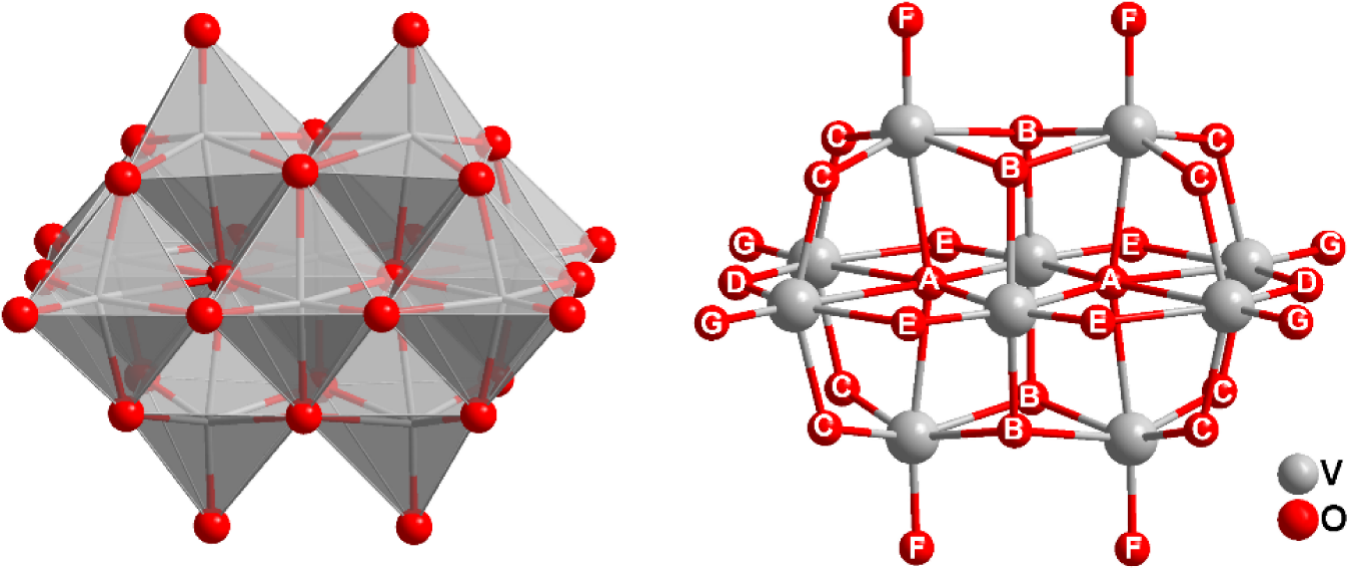
Polyhedral representation and ball and stick structure of [V_10_O_28_]^6–^, representing the different types of oxygen atoms (O_A_ (−μ_6_O), O_B_ (−μ_3_O), O_C_ (−μ_2_O), O_D_ (−μ_2_O), O_E_ (−μ_2_O), O_F_ (V=O) and O_G_ (V=O)).

Noncovalent interactions between V_10_ and organic cations have been studied using computational chemistry methods. Recently, Paredes-Pérez et al.^19^ investigated the interactions between V_10_ and polyamine cations using Quantum Theory of Atoms in Molecules (QTAIM)^18^ and Noncovalent Interaction-Reduced Density Gradient (NCI-RDG) analyses.^200^ Studies with both organic and inorganic cations have demonstrated that electrostatic forces, hydrogen bonding and other noncovalent intermolecular interactions play a key role in their crystal lattices.^20,21^ Oxygen atoms on the surface of V_10_ are known to provide multiple sites to form coordinate bonds with s- and d-block metal cations, making V_10_ a key building block for constructing well-defined supramolecular structures. It was also demonstrated that the structure of some of these bimetallic structures remains intact in aqueous and buffered media.^22^ However, there is a lack of systematic rational approaches to synthesize these heterometallic species, and the understanding of the self-assembly process, along with the factors involved in TM-V_10_ bond stabilization, is limited.

The structure of V_10_ consists of ten {VO_6_} edge- and corner-sharing octahedra (Figure 1), resulting in an approximate D_2h_ symmetry. It was demonstrated by Density Functional Theory (DFT) that V_10_ has a highly negative surface with seven nonequivalent oxygen atoms, categorized based on their coordination modes and the position of the vanadium centers in the aggregate as O_A_ (−μ_6_O), O_B_ (−μ_3_O), O_C_ (−μ_2_O), O_D_ (−μ_2_O), O_E_ (−μ_2_O), O_F_ (V=O) and O_G_ (V=O). Among these, the O_B_ and O_C_ sites are the most basic, as indicated by electrostatic surface potential (ESP) calculations, and are regarded as the most probable sites for protonation.^23,24^ Although the protonation of these nucleophilic oxygen sites was experimentally confirmed, the same trend is not observed when V_10_ acts as a ligand for metal complexes (Table S1). V_10_ typically binds weakly to TM ions, primarily through O_C_ and O_D_, followed by O_F_ and O_G_, while the O_B_ site is known to be constrained due to steric hindrance.^25^

This study focuses on the cyclen macrocycle system as a model to assess the factors influencing the coordination of V_10_ to a second transition-metal ion. Therefore, three new hybrid compounds based on TM–cyclen cations, where TM = nickel­(II), copper­(II) and zinc­(II), were synthesized, giving an ionic pair in [Ni­(cyclen)­(H_2_O)_2_]_2_[H_2_V_10_O_28_]·2H_2_O (**1**), and decorated V_10_ structures in [{Cu­(cyclen)}_2_(H_2_V_10_O_28_)]·9H_2_O (**2**) and [{Zn­(cyclen)}_3_(V_10_O_28_)]·4H_2_O (**3**). Among over 40 TM–V_10_ structures deposited in the Cambridge Crystallographic Data Centre (Table S1), complexes **2** and **3** are the first examples of this polyoxoanion acting as a ligand through the O_B_ atoms, and **3** is the only one in which V_10_ is decorated with three complexes. These three hybrid compounds were used as models to computationally investigate the effect of noncovalent interactions on the coordination behavior of V_10_. The electrostatic attraction between V_10_, acting as a Lewis base, and the {TM­(cyclen)}^2+^ Lewis acids can be described in terms of a region of electron depletion on the TM complexes that is oriented perpendicular to the TM–V_10_ bond plane—a feature known as π-hole.^26–28^ Recent studies have shown that metalloporphyrins containing Co­(II), Cu­(II), or Zn­(II) display π-holes centered on the metal ion (i.e., a positive ESP above/below the porphyrin plane), whereas an isostructural Ni­(II) complex does not,^29^ highlighting the challenge of predicting this behavior. π-Holes, due to their highly directional character, have also been shown to play a crucial role in rationalizing semicoordination and other noncovalent interactions, with importance in biological systems^30^ and the design of new materials.^31–33^ Therefore, understanding this electrostatic feature is essential for predicting and explaining the directional interactions between cyclen-based complexes and V_10_, which are necessary for the progress of supramolecular chemistry.^34^ Moreover, to elucidate the contributions of both covalent and noncovalent forces in the formation of the new hybrid materials, the Intrinsic Bond Strength Index (IBSI)^35^ was calculated in combination with the Independent Gradient Model based on Hirshfeld partitioning of the molecular density (IGMH).^36^

Furthermore, POMs and POVs are recognized for their effectiveness in treating various wastewater effluents, functioning as photocatalysts for the degradation of pollutants,^37^ as flocculating agents,^38^ or as adsorbent materials for a range of hazardous substances.^39–41^ Specifically, methylene blue (MB^+^) is a thiazine dye abundantly found in textile industry effluents that is nonbiodegradable and capable of causing serious health and environmental problems.^42^ The dye-bleaching of MB^+^ by POM or POM-based hybrid organic–inorganic materials is often attributed to cation-exchange or adsorption-driven mechanisms involving the electrostatic interactions between the high negative surface charge of POMs and organic cations.^43–49^ For instance, the adsorption efficiency of MB^+^ increases for Keggin vanadium-substituted polyoxomolybdates as the V­(V):Mo­(VI) proportion changes from 1:11 to 3:9, modulating the charge density of the material.^50^ Decavanadate-based compounds, such as (2-ampH)_6_[V_10_O_28_][Bibr ref51] (2,3-ampH)_6_[V_10_O_28_]·4H_2_O^52^ and [Cu­(H_2_O)_3_(2–amp)]_2_(trisH)_2_[V_10_O_28_]·2H_2_O,^53^ were also found to efficiently bleach MB^+^ solutions. Moreover, in the past few years, efforts were made to better understand the intermolecular interactions of some cationic organic dyes with V_10_ and other POVs,^51,54,55^ exploring supramolecular features,^52,56^ medicinal properties^57^ and the effects on the optical properties of the dyes.^43,52,57–61^ For instance, the interaction of rhodamine B (RB^+^) and MB^+^ with V_10_ in aqueous solution is governed by strong electrostatic interactions, rendering crystals of the hybrid organic–inorganic salts (RB)_4_[H_2_V_10_O_28_]·2RB^57^ and (MB)_4_[H_2_V_10_O_28_].^52^ Inspired by our previous studies,^52,53^ compounds **1** to **3** were employed to explore how variations in structures and surface charge distribution in POVs affect their efficiency to bleach MB^+^ solutions. The distinct effectiveness was rationalized by combining molecular modeling, morphological studies, and supramolecular structural patterns analysis.

## Experimental Section

### General Information

The reactions were carried out in ultrapure water (type 3, resistivity <3.5 μS cm^–1^ at 25 °C) at ambient conditions in air. The chemical reagents CuCl_2_·2H_2_O, NiCl_2_, ZnCl_2_, NaVO_3_ (≥97.0%, Aldrich), 1,4,7,10-tetraazacyclododecane (cyclen, 98%, Apollo Scientific), methylene blue (MB^+^, 3,7-bis­(dimethylamino)-phenothiazin-5-ium chloride, 97,0%, Aldrich), H_2_O_2_ (32,5%) and HCl (37%) were used without further purification. Carbon, hydrogen, and nitrogen contents were determined on a PerkinElmer 2400 Series elemental analyzer. The Fourier-transformed infrared (FTIR) spectra were obtained from KBr pellets on a VERTEX 70v spectrophotometer, with a resolution of 2 cm^–1^ within the range of 400 to 4000 cm^–1^. The thermogravimetric analysis (TGA) was performed from 25 to 900 °C at a rate of 10 °C min^–1^ on a Netzsch STA449 F3 Jupiter thermobalance under synthetic air. Powder X-ray diffraction (PXRD) patterns were collected in a Shimadzu XRD-600 diffractometer operating at 30 kV/20 mA equipped with Cu–Kα radiation (λ = 1.5418 Å). Electron paramagnetic resonance (EPR) spectra were recorded from pulverized solid samples at room temperature and at 77 K on a Bruker ELEXSYS MX-micro spectrometer operating in X-band (9.5 GHz). The Scanning Electron Microscopy (SEM) analyses were carried out in a TESCAN VEGA3 LMU equipment at different magnifications, with a resolution of 3 nm. EDS (Energy Dispersive Spectroscopy) spectra were obtained using a chemical analysis system (Oxford) with AZ Tech software (Advanced) with an 80 mm^2^ SDD-type detector. The mass spectrum was obtained using a Thermo Scientific LTQ-XL ion trap spectrometer, with electrospray ionization and quadrupole mass analyzer. The N_2_ physisorption isotherm of product **2** was measured at −196 °C in a Nova 2000e equipment from Quantachrome Instruments after degassing the sample at 100 °C under vacuum for 16 h. The specific surface area was obtained following the Brunauer–Emmett–Teller (BET) method, the micropore volume and external surface area were calculated by the t-plot method and the total pore volume was determined at a relative pressure of 0.98 by following Gurvich’s approach.

### Synthetic Procedures

#### Synthesis of [Ni­(cyclen)­(H_2_O)_2_]_2_[H_2_V_10_O_28_]·4H_2_O (**1**)

A suspension of NaVO_3_ (304.8 mg, 2.499 mmol) in 50 mL of water was heated at 70 °C for 20 min, rendering a colorless solution. After cooling to room temperature, the pH was adjusted to 5.0 with diluted HCl, forming an orange solution. Subsequently, a purple solution containing 118.85 mg (0.5 mmol) of NiCl_2_·6H_2_O and 86.1 mg (0.499 mmol) of cyclen in 20 mL of water was added under magnetic stirring. The pH of the reaction mixture was adjusted to 5.0 with dilute HCl. After 3 days on the bench, brown rhombus-shaped crystals were isolated by filtration, washed with 100 mL of water, and dried in air. The solid was insoluble in water and in both polar and nonpolar organic solvents. Yield: 0.3028 g, 79% based on vanadium (C_16_H_58_O_36_N_8_Ni_2_V_10_). FTIR (KBr, cm^–1^): 3323­(br), 3233­(w), 1618­(w), 1454­(w), 1308­(w), 1110­(w), 960(s), 831(s), 748(s), 611­(m), 548­(m) (w = weak, s = strong, br = broad and m = medium). Elemental analysis: calculated (%) for (C_16_H_58_N_8_O_36_V_10_Ni_2_): C, 12.3; H, 3.73; N, 7.16. Found (%) for 1: C, 12.7; H, 3.54; N, 7.25. Based on elemental analysis and TGA data, there is a difference in the crystallization water content between the single crystals and the bulk material (two versus four water molecules, respectively). See discussion below.

### Synthesis of [{Cu­(cyclen)}_2_(H_2_V_10_O_28_)]·9H_2_O (**2**)

NaVO_3_ (304.8 mg, 2.499 mmol) was solubilized in boiling water (40 mL) under stirring. The resulting colorless solution turned orange after its pH was adjusted to 5.0 using dilute HCl. A 20 mL aqueous solution containing CuCl_2_·2H_2_O (0.0852 g, 0.5 mmol) and cyclen (0.0861 g, 0.5 mmol) was added dropwise to the orange reaction mixture at room temperature, and a dark green solid immediately precipitated. After 3 days, the solid was filtered off and air-dried. The product was insoluble in water and in both polar and nonpolar organic solvents. Yield: 0.2973 g, 78% based on vanadium (C_16_H_60_O_37_N_8_Cu_2_V_10_). FTIR (KBr, cm^–1^): 3424­(br), 3279­(w), 3227­(w), 1012­(w), 961(s), 836(s), 746(s), 606­(m), 549­(m), 432­(w) (w = weak, s = strong, br = broad and m = medium). Elemental analysis: calculated (%) for C_16_H_60_O_37_N_8_Cu_2_V_10_: C, 12.1; H, 3.80; N, 7.03. Found (%) for 2: C, 12.2; H, 3.43; N, 7.04. Crystals suitable for single-crystal X-ray diffraction analysis were obtained from a 10-time diluted reaction mixture from the beaker wall after several attempts.

### Synthesis of [{Zn­(cyclen)}_3_(V_10_O_28_)]·4H_2_O (**3**)

A suspension of NaVO_3_ (304.8 mg, 2.499 mmol) was dissolved in boiling water (40 mL) under stirring. After reaching room temperature, the resulting colorless solution was acidified to pH 4.0 with dilute HNO_3_, producing an orange solution. A 20 mL aqueous system containing cyclen (0.54 mmol, 94.3 mg) and ZnCl_2_ (0.499 mmol, 68.0 mg) was then gradually added to the decavanadate solution, leading to the immediate formation of a precipitate. After 2 days, prismatic orange crystals were obtained. The solid was gently filtered, washed with water, and air-dried. It was partially soluble in water, DMSO, THF, methanol, chloroform, and insoluble in toluene and ethyl acetate. Yield: 0.4965 g, 28% based on vanadium (C_24_H_68_O_32_N_12_Zn_3_V_10_). FTIR (KBr, cm^–1^): 3568­(br), 3259­(w), 1609­(w), 1442­(w), 1277­(w), 1091­(w), 963(s), 826(s), 743(s), 588­(m), 433­(m) (w = weak, s = strong, br = broad and m = medium). Elemental analysis: calculated (%) for (C_24_H_68_O_32_N_12_Zn_3_V_10_): C, 16.5; H, 3.93; N, 9.65. Found (%) for 3: C, 16.1; H, 3.67; N, 9.29.

### Single-Crystal X-ray Diffraction Analysis (SC-XRD)

Single-crystal X-ray diffraction data for compounds **1** and **3** were collected using a Bruker D8 Venture diffractometer equipped with a Photon 100 CMOS detector, employing Mo–Kα radiation (λ = 0.71073 Å) and a graphite monochromator. Data acquisition was carried out via thin-slice ω and φ scans. Data integration and reduction were performed with the APEX3 software package.^62^

For compound **2**, intensity data were collected using a Bruker D8 Venture diffractometer equipped with an Incoatec IμS 3.0 microfocus source (Cu–Kα radiation, λ = 1.54178 Å) and a Photon III CPAD detector. In this case, data collection, reduction, and absorption correction were carried out using the APEX4 software suite.^63^

Data for compound **1** was collected at 300 K, while for **2** and **3**, measurements were performed at 100 K. In all cases, multiscan absorption corrections were applied using SADABS v2.03.^64^

The crystal structures were solved by direct methods using the SIR-2004 software^65^ and refined by full-matrix least-squares on F^2^ using SHELX2018/3.^66^ For compound **2**, multiple data sets were collected using different temperatures and both Mo–Kα and Cu–Kα radiations in order to locate the water molecules more precisely (see below). The structure reported herein corresponds to the data set providing the highest-quality data.

In all three structures, non-hydrogen atoms were refined anisotropically. Hydrogen atoms bonded to nitrogen atoms of the cyclen macrocycle were identified from Fourier difference maps and freely refined. Hydrogen atoms bonded to carbon atoms within the cyclen framework were positioned geometrically and refined using a riding model. For compounds **1** and **2**, the hydrogen atom of the HV_5_O_14_ moiety (present as one-half of the [H_2_V_10_O_28_]^2–^ cation in the asymmetric unit) was located in the Fourier map and refined accordingly.

Regarding water molecules, hydrogen atoms were located and refined for compounds **1** and **3**; the distances O–H and H···H in the water molecules were set, in accordance with the collection temperatures, by using the DFIX and DANG restraints in SHELXL. All hydrogen atoms observed in the Fourier difference maps were freely refined, with thermal parameters constrained relative to their parent atoms. For compound **2**, no water hydrogen atoms could be resolved; thus, solvent contributions were treated using the SQUEEZE routine implemented in PLATON, and the structural model was refined accordingly. The analysis performed with SQUEEZE revealed the presence of 180 electrons per unit cell which were assigned to 18 water molecules (i.e., 9 for every [Cu­(C_8_H_20_N_4_)]_2_[H_2_V_10_O_28_] unit).

The crystallographic data and refinement parameters of **1**, **2** and **3** are listed in Table 1, and the crystallographic data are available in Tables S2 – S8.

**Table 1:** Crystallographic Data and Refinement Parameters for Compounds **1**–**3**

	1	2	3
Formula	[Ni(C_8_H_20_N_4_)(H_2_O)_2_]_2_[H_2_V_10_O_28_][H_2_O]_2_	[Cu(C_8_H_20_N_4_)]_2_[H_2_V_10_O_28_][H_2_O]_9_	[Zn(C_8_H_20_N_4_)]_3_[V_10_O_28_][H_2_O]_4_
MW	1529.48	1593.22	1742.41
T (K)	300	100	100
λ (Å)	0.71073	1.54178	0.71073
Crystal system, space group	Monoclinic, P2_1_/n	Monoclinic, P2_1_/n	Monoclinic, *Cc*
Unit cell dimensions (Å, °)	a = 13.2041(7)	a = 10.9580(6)	a = 19.9132(10)
	b = 8.8076(5), β = 102.412(2)	b = 16.0613(9); β = 101.202(3)	b = 12.9809(6); β = 105.489(2)
	c = 19.5243(10)	c = 13.4016(8)	c = 21.2103(10)
Volume (Å^3^)	2217.5(2)	2313.7(2)	5283.6(4)
Z, dc (mg/cm^3^)	2, 2.291	2, 2.552	4, 2.190
μ (mm^–1^)	2.942	18.157	3.117
F(000)	1528	1592	3488
Crystal size	0.578 × 0.292 × 0.110	0.35 × 0.28 × 0.20	0.332 × 0.270 × 0.194
Reflns collected/unique (Rint)	83813/5521 (0.0306)	17312/3901 (0.0929)	213547/12723 (0.0372)
2θ range (°)	5.602/56.666	8.692/130.636	5.072/55.998
Data/parameters	5521/349	3901/304	12723/791
Final R indices [I > 2σ]	R1 = 0.0238, wR2 = 0.0557	R1 = 0.0531, wR2 = 0.1320	R1 = 0.0238, wR2 = 0.0570
R indices (all data)	R1 = 0.0306, wR2 = 0.0603	R1 = 0.0711, wR2 = 0.1438	R1 = 0.0282, wR2 = 0.0609
GoF	1.095	0.993	0.910

### Theoretical Calculations

All quantum chemistry calculations were conducted employing the Density Functional Theory (DFT) computational method using the ωB97X-D3 functional^67,68^ and def2-tzvp basis set,^69^ as implemented in the Orca 5.0.4 software package.^70^ This theory level was selected due to its well-documented performance in the description of weak noncovalent interactions, including dispersion-dominated and hydrogen-bonded complexes.^71–73^ The optimized geometries for the molecular models of compounds **2** and **3**, the isolated {TM­(cyclen)}^2+^ fragments (TM = Ni, Cu, Zn) (Tables S12–S14), and the decavanadate anions were calculated in vacuum using the crystallographic data from the SC-XRD analysis as the starting point. The optimized structures were subjected to vibrational frequency calculations and the absence of imaginary frequencies confirmed that they are potential energy surface minima. Electrostatic surface potential (ESP) maps, Intrinsic Bond Strength Index (IBSI), and Independent Gradient Model based on Hirshfeld partition (IGMH) analyses were conducted using the Multiwfn 3.8 software.^74–76^ The isosurfaces were rendered using the VMD—Visual Molecular Dynamics 1.9.3 software.^77^ The frontier orbitals were rendered by Chemcraft 1.8.^78^ The IBSI values were calculated using the same theory level as the reference value for H_2_ (bond length: 0.7400 Å). Charge Decomposition Analysis (CDA) and frontier molecular orbital analysis were also performed to investigate orbital contributions. EPR parameters were calculated by DFT and plotted using EasySpin 4.0.0.^79^

### Methylene Blue Adsorption Experiments

In a typical experiment, 10.0 mg of **1**, **2**, or **3** were directly added to 100 mL of a 10 mg L^–1^ aqueous solution of MB^+^ under magnetic stirring to ensure homogeneity. Aliquots of 4.0 mL of the reaction medium were taken at 2, 5, 10, 15, 20, 25, 30, 35, and 40 min after the beginning of the reactions. Each aliquot was centrifuged at 8000 rpm for 1 min and the supernatant was carefully removed for subsequent UV–vis analysis, monitoring the intensity of the band at 664 nm. Additional experiments were performed with compound **2**, in which decreasing amounts (2, 4, 6, and 8 mg) were tested. The bleaching capacity of the POVs was calculated using the formula % = (A_0_ – A)/A_0_ × 100, wherein A_0_ is the absorbance of the dye solution before the reaction and A is the absorbance of the reaction solution at any time point.^52,53^ All experiments were run at least in triplicate, and the results are expressed as arithmetic averages, considering mean ± standard deviation (SD).

## Results and Discussion

### Synthesis and Crystallography (Structural Characterization)

The synthetic strategy employed to form the hybrid products was based on the independent preparation of V_10_ and the TM-cyclen cationic complexes, which act as building blocks for the bimetallic species. The syntheses were carried out in water, wherein an orange solution of sodium decavanadate received the slow addition of the chloride salt solutions of [Ni­(H_2_O)_2_(cyclen)]^2+^ (violet), [Cu­(H_2_O)­(cyclen)]^2+^ (blue), or [Zn­(H_2_O)­(cyclen)]^2+^ (colorless) in 1 V_10_:2 TM-cyclen proportion. Under similar conditions, the reaction containing nickel­(II) yielded brown crystals (**1**), while the reactions with copper­(II) and zinc­(II) immediately formed green (**2**) and orange precipitates (**3**), respectively. For **2** and **3**, crystals suitable for SC-XRD were obtained from 10-time diluted reactions. Both products of the most concentrated and most dilute syntheses exhibited similar profiles when analyzed by PXRD and spectroscopic analysis, differing only in the size of the crystallites (See discussion below).

### Crystal Structure Description of [Ni­(cyclen)­(H_2_O)_2_]_2_[H_2_V_10_O_28_]·2H_2_O (**1**)

Compound **1** crystallizes in the monoclinic P2_1_/n space group. The asymmetric unit of 1 consists of one-half of a deprotonated [H_2_V_10_O_28_]^4–^ anion, one [Ni­(H_2_O)_2_(cyclen)]^2+^ cation complex and one crystallization water molecule (Figure 2a). The resulting salt can be formulated as [Ni­(H_2_O)_2_­(cyclen)]_2_­[H_2_­V_10_­O_28_]·2H_2_O. The protonation of V_10_ occurs on two O_C_ (−μ_2_O) oxygen atoms (O3 in Figure 2a). The V–O bond lengths and angles in the polyoxoanion are consistent with other decavanadate clusters reported in the literature.^80,81^ Bond valence sum (BVS) calculations^82^ for the oxygen atoms of V_10_ resulted in a value of 1.209 for bridging O(3), O_C_ in Figure 1, within the range of 1.2 to 1.5 reported for protonated oxygen atoms in other decavanadate structures.^83^ As expected, for the nonprotonated oxygen atoms, the BVS values range from 1.76 to 1.95 (Table S8). Regarding the [Ni­(H_2_O)_2_(cyclen)]^2+^ complex cation, the macrocycle binds the metal ion, leaving a *cis* vacancy occupied by two water molecules with bond lengths Ni–O­(1W) of 2.128(2) and Ni–O­(2W) of 2.118(2) Å. Overall, the resulting octahedral geometry is significantly distorted, as evidenced by the N–Ni–N bond angles, which show substantial deviations from the values expected for an ideal octahedron. Moreover, the angle O­(1W)–Ni–O­(2W) of 81.72(8)° also reflects the restrictions imposed by cyclen. This is a usual coordination mode of cyclen to nickel­(II) in complexes with other mono and bidentate ligands.^84–87^

**Figure 2:**
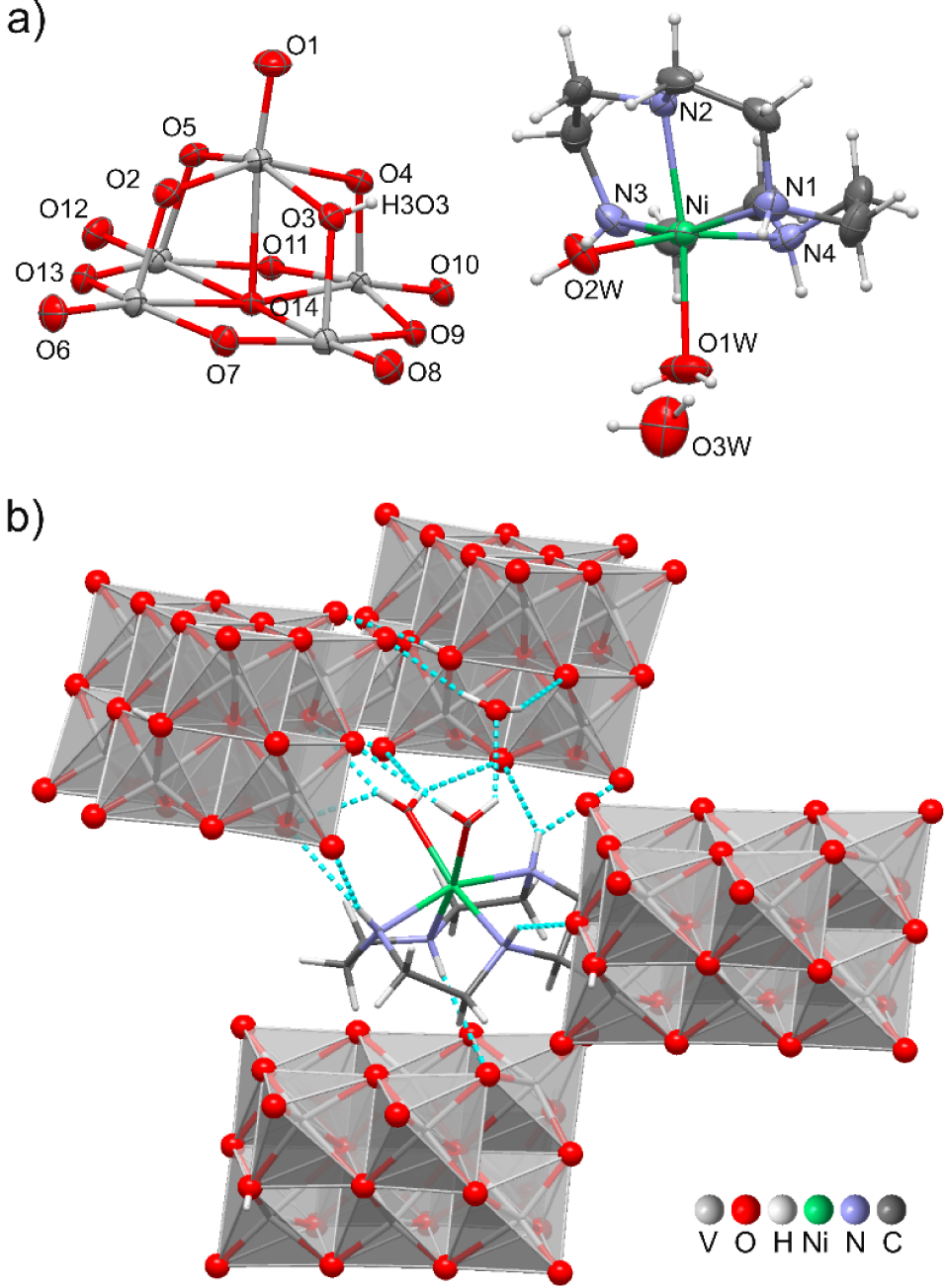
(a) Asymmetric unit of [Ni­(H_2_O)_2_(cyclen)]_2_[H_2_V_10_O_28_]·2H_2_O (**1**), indicating the atom numbering scheme (a selection). Thermal ellipsoid representation at 50% probability. (b) Intermolecular N–H···O hydrogen bond interactions, represented as turquoise dashed lines, between cyclen and four V_10_ anions.

An extensive hydrogen bond network involving cyclen, water molecules, and the terminal and bridging oxygen atoms of [H_2_V_10_O_28_]^4–^ anions plays a significant role in stabilizing the crystal packing (Table 2; only NH···O hydrogen bonds were considered, with H···O distances shorter than the sum of the H and O van der Waals radii, and NHO angles larger than 130°). Based on the geometrical parameters which define the H-bonds, the strongest hydrogen bond involves O(3)–H­(3O3)···O(8) (O_F_ in Figure 1). As a result, a chain of V_10_ anions (hold together by 4 identical O–H···O hydrogen bonds) originates which extends along the *b*-axis direction (Figure S1). The crystallization water molecule O­(3W), which works as H-bond donor toward two symmetry related V_10_ further contributes to reinforce the V_10_ chain, as well as one of the Ni­(II)-coordinated water molecule (O2W) which is involved in weak^88^ O–H···O hydrogen bonds with two symmetry related V_10_ (each hydrogen atom acts as a bifurcated donor; the H···O distances range from 2.19(2) to 2.53(1) Å, and O–H···O angles between 133(1)° and 156.5(0.9)°). Cyclen nitrogen atoms participate in six weak N–H···O hydrogen bonds with four V_10_ provided by three adjacent chains, with H···O distances ranging from 2.30(3) to 2.61(3) Å and N–H···O angles between 132(2)° and 169(3)° (Figure 2b). Overall, the crystal structure exhibits a layered arrangement composed of decavanadate chains and Ni­(II)-cyclen complexes (replicated along the *b*-axis direction) which alternate along the *c*-axis (Figure S2).

**Table 2:** Selected Hydrogen Bonds Observed in **1**, **2** and **3** ^a^

D–H···A	d(D–H) Å	d(H···A) Å	d(D···A) Å	<(DHA) °
Compound **1**				
N(1)–H(1)···O(6)#1	0.83(0.03)	2.57(0.03)	3.186(0.003)	132(2)
N(1)–H(1)···O(7)#1	0.83(0.03)	2.61 (0.03)	3.220(0.002)	132(2)
N(2)–H(2)···O(5)#2	0.83(0.03)	2.45(0.03)	3.279(0.002)	169(3)
N(3)–H(3)···O(6)#3	0.76(0.03)	2.52 (0.03)	3.206(0.003	149(3)
N(3)–H(3)···O(10)	0.76(0.03)	2.49 (0.03)	3.118(0.003)	141(3)
N(4)–H(4)···O(2)#4	0.88(0.03)	2.30(0.03)	3.098(0.003)	151(3)
O(3)-H(3O3)···O(8)#1	0.65(0.03)	2.10(0.03)	2.745(0.002)	169(3)
O(1W)-H(1WA)···O(13)#3	0.82(0.02)	2.30(0.02)	3.117(0.003)	176(2)
O(1W)-H(1WB)···O(3W)	0.82(0.02)	2.16(0.02)	2.775(0.003)	132(2)
O(2W)-H(2WA)···O(10)	0.82(0.01)	2.48(0.01)	3.095(0.002)	133(1)
O(2W)-H(2WA)···O(9)	0.82(0.01)	2.19(0.02)	2.953(0.002)	154 (2)
O(2W)-H(2WB)···O(8)#1	0.82(0.01)	2.35(0.03)	2.994(0.003)	136(1)
O(2W)-H(2WB)···O(7)#1	0.82(0.01)	2.53(0.01)	3.295(0.002)	156.5(0.9)
O(3W)-H(3WA)···O(2)#1	0.82(0.01)	2.17 (0.01)	2.956(0.003)	161.6(0.9)
O(3W)-H(3WB)···O(12)#3	0.82(0.03)	2.18(0.02)	2.941(0.003)	154(2)
Compound **2**				
N(1)-H(1N1)···O(9)#1	0.91(0.06)	2.03(0.06)	2.903(0.006)	162(5)
N(2)-H(1N2)···O(6)	0.82 (0.07)	2.70(0.06)	3.320(0.006)	133(5)
N(3)-H(1N3)···O(5)	0.75(0.06)	2.25(0.07)	2.915(0.006)	147(6)
N(4)-H(1N4)···O(8)#1	0.84(0.06)	2.40((0.07)	3.021(0.006)	131(5)
N(4)-H(1N4)···O(12)	0.84(0.06)	2.63(0.06)	3.266(0.006)	133 (5)
Compound **3**				
N(1A)–H(1A)···O(12A)	0.70(0.07)	2.54(0.07)	3.108(0.006)	139(8))
N(2A)–H(2A)···O(5A)	0.93(0.06)	2.04(0.06)	2.879(0.005)	149(5)
N(3A)–H(3A)···O(6A)	0.80(0.07)	2.37(0.06)	3.008(0.006)	138(6)
N(4A)–H(4A)···O(9B)	0.79(0.08)	2.33(0.07)	2.900(0.005)	130(6)
N(2B)–H(2B)···O(9A)	0.86(0.06)	2.10(0.06)	2.870(0.006)	149(6)
N(4B)–H(4B)···O(5B)	0.71(0.06)	2.31(0.06)	2.935(0.005)	148(6)
N(1C)–H(1C)···O(2B)	0.86(0.07)	2.38(0.08)	3.004(0.006)	130(6)
N(3C)–H(3C)···O(12A)	0.88(0.04)	2.20(0.05)	2.978(0.005)	147(5)
N(4C)–H(4C)···O(3B)	0.89(0.08)	2.22(0.07)	2.890(0.005)	132(6)
N(1A)–H(1A)···O(3W3)#1	0.70(0.07)	2.50(0.07)	3.015(0.006)	132(8)
N(3A)–H(3A)···O(4W4)	0.80(0.07)	2.58(0.07)	3.268(0.006)	145(6)
O(1W1)–H(1W1)···O(3A)#2	0.85(0.04)	1.96(0.04)	2.799(0.005)	171(4)
O(1W1)–H(2W1)···O(1B)	0.84(0.04)	1.93 (0.04)	2.768(0.005)	177(4)
O(2W2)–H(1W2)···O(12A)#3	0.85(0.05)	2.11(0.05)	2.908(0.005)	157(5)
O(2W2)–H(2W2)···O(1W1)	0.85(0.04)	1.89(0.03)	2.719(0.006)	167(6)
O(3W3)–H(1W3)···O(2W2)	0.84(0.04)	1.97(0.05)	2.792(0.006)	165(4)
O(3W3)–H(2W3)···O(4W4)#2	0.84(0.04)	2.08(0.05)	2.886(0.006)	161(5)
O(4W4)–H(2W4)···O(10A)#4	0.84(0.03)	2.05(0.02)	2.874(0.006)	169(5)

a Symmetry transformations used to generate equivalent atoms: For **1**: #1: 1–x, 1–y, 1–z; #2: 1.5–x, 0.5+y, 1.5–z; #3: 1–x, -y, 1–z; #4: −0.5+x, 0.5–y, 0.5+z; For **2**: #1: −x+1, −y+1, −z+1; For **3**: #1: x, 1–y, −0.5+z; #2: 0.5+x, 0.5–y, 0.5+z; #3: x, 1–y, 0.5+z; #4: x, −1+y, +z.

### Crystal Structure Description of [{Cu­(cyclen)}_2_(H_2_V_10_O_28_)]·9H_2_O (**2**)

The crystals of **2** belong to the monoclinic P2_1_/n space group. The asymmetric unit of **2** reveals the presence of the {Cu­(cyclen)­(HV_5_O_14_)} (Figure 3a), being the other half of this neutral species generated by symmetry. In addition, highly disordered solvent molecules are present which, based on the residual electron density estimated with the SQUEEZE method, have been identified as 18 water molecules in the unit cell. Hence, the formula is [{Cu­(cyclen)}_2_(H_2_V_10_O_28_)]·9H_2_O, where the V_10_ acts as a bridging ligand between two {Cu­(cyclen)}^2+^ fragments [Cu–O(13) 2.253(4) Å]. To the best of our knowledge, this is the first time that this POV acts as a ligand of a transition metal ion through the triply bridging oxygen atom (O_B_); see Table S1 and additional discussion presented below. V_10_ is deprotonated on the O(11) doubly bridging oxygen atoms (O_C_ in Figure 1) with an O–H distance of 1.02(3) Å. The BVS calculations^82^ are in the range of 1.61 and 1.95 for all oxygen atoms, except for the O(11), which exhibits a BVS of 1.41 (Table S9).

**Figure 3:**
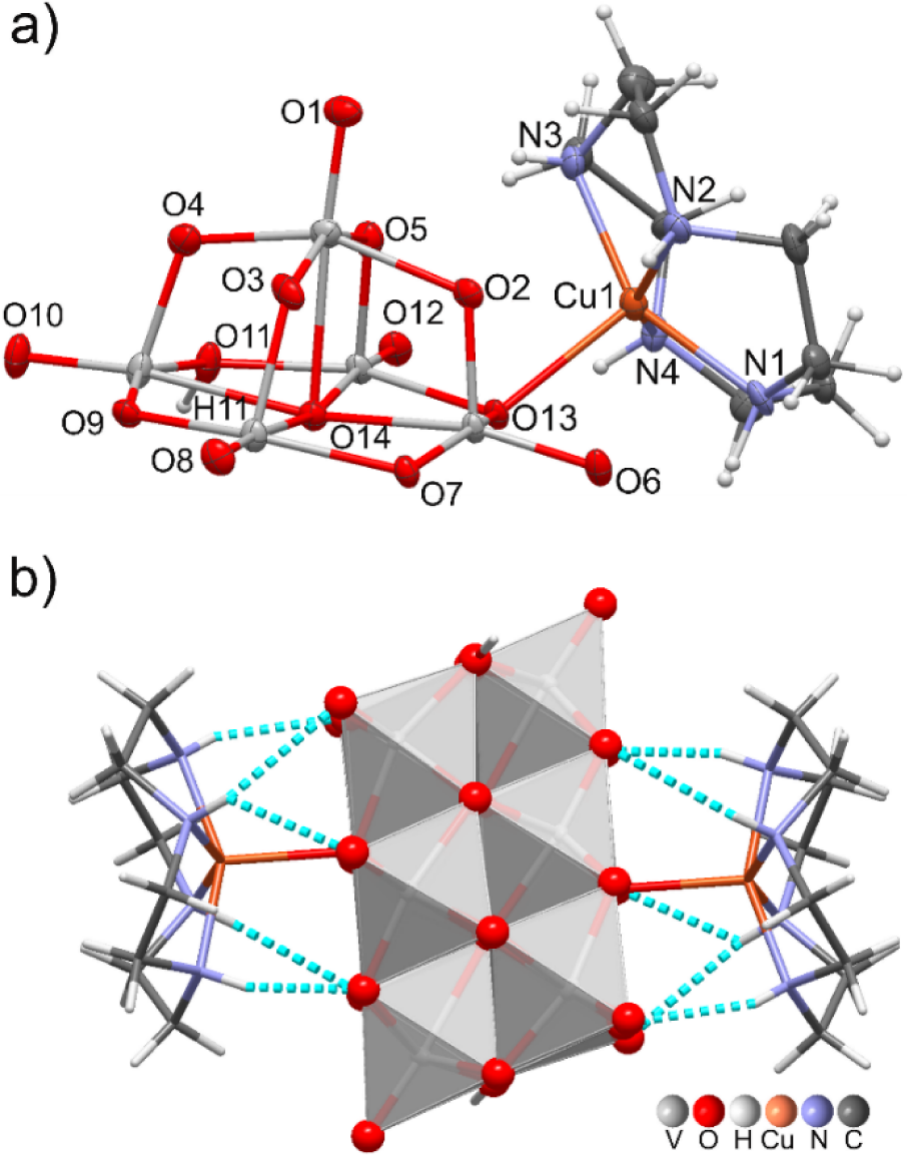
(a) Asymmetric unit of [{Cu­(cyclen)}_2_(H_2_V_10_O_28_)]·9H_2_O (**2**), indicating the atom numbering scheme (a selection). Thermal ellipsoid representation at 50% probability. (b) Intramolecular N–H···O hydrogen bond interactions, represented as turquoise dashed lines, between the TM-cyclen and the V_10_ anion.

The copper­(II) coordination geometry is close to a distorted square pyramid, with the four nitrogen atoms of cyclen defining the basal plane, and O(13) of V_10_ occupying the axial position. This configuration results in an inverted umbrella arrangement^89^ (Figure 3a), where the distance from Cu to the basal plane of 0.52 Å falls within the expected range (0.48 to 0.62 Å) reported for other Cu-cyclen complexes.^90^

Each {Cu­(cyclen)}^2+^ fragment also interacts with {V_10_}^4–^ through five intramolecular hydrogen bonds involving 2 O_F_, 2 O_C_ and 1 O_D_ V_10_-oxygen atoms. NH···O distances ranging from 2.03(6) to 2.70(6) Å and N–H···O angles from 131(5) to 162(5)° (Figure 3b, and Table 2). The crystal packing viewed along the *a*-axis direction evidence voids (13.8% of the unit cell volume; probe radius 1.2 Å, approximated grid 0.7 Å) which most probably host the solvent water molecules (Figure S3). These latter together with several quite weak intermolecular contacts, contribute to stabilize the overall crystal architecture.

### Crystal Structure Description of [{Zn­(cyclen)}_3_(V_10_O_28_)]·4H_2_O (**3**)

Compound **3** crystallizes in the monoclinic *Cc* space group, and its asymmetric units consist of one crystallographically independent [{Zn­(cyclen)}_3_(V_10_O_28_)] molecule along with four crystallization water molecules (Figure 4a). One interesting feature of this structure is the presence of a nonprotonated decavanadate subunit {V_10_}^6–^ acting as a bridging ligand of three {Zn­(cyclen)}^2+^ fragments by triple-bridging oxygen O_B_ atoms with Zn­(1A)–(13A) Zn­(1B)–O­(13B) and Zn­(1C)–O­(7B) bond lengths of 1.992(3), 1.973(3) and 1.997(3) Å. Notably, the discrete structure of 3, containing V_10_ decorated with more than two metal complexes, is unprecedent. The Zn­(II) centers adopt a distorted square pyramidal geometry, with the metal ion located at a distance of approximately 0.81 Å above the basal plane formed by the four nitrogen atoms of each cyclen. The framework is further stabilized by intramolecular N–H···O hydrogen bonds between each Zn-cyclen complex and V_10_ anion (Table 2 and Figure 4b): four for Zn­(1A)-cyclen-, three for Zn­(1C)-cyclen- and two for Zn­(1B)-cyclen-V_10_ species, respectively. Bond distances (NH···O) and angles range from 2.04(6) and 2.54(7) Å, and 130(6) and 149(6)^o^, respectively. In particular, 1 O_F_, 2 O_D_ and 4 O_C_ atom types are involved, with the O_F_ one acting as acceptor toward two Zn­(cyclen) complexes and a crystallization water molecule. The four water molecules are interconnected (with O1W1 and O4W4 being the terminal ones in the chain), and each forms three hydrogen bonds (Table 2 and Figure S4). They act as hydrogen bond donors toward the oxygen atoms O_C_ (O1W1/O3A), O_F_ (O2W2/O12A), and O_G_ (O1W1/O1B; O4W4/O10A) provided by different V_10_ units, and as acceptors from the NH groups of symmetry related cyclen ligands (O3W3 and O4W4).

**Figure 4:**
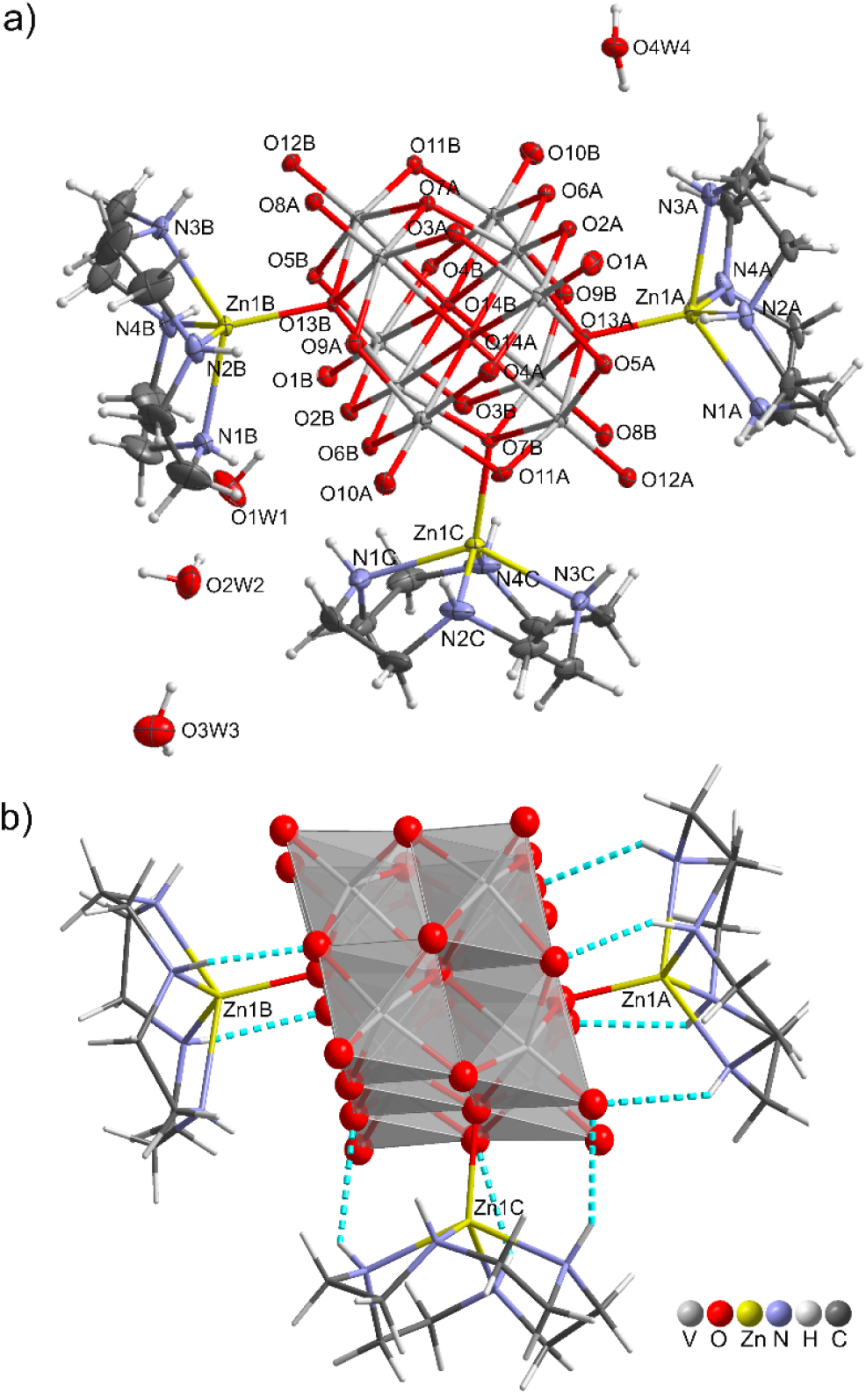
(a) Asymmetric unit of [{Zn­(cyclen)}_3_(V_10_O_28_)]·4H_2_O (**3**) with thermal ellipsoid representation at 50% probability for non-hydrogen atom. (b) Intramolecular N–H···O hydrogen bond interactions, represented as turquoise dashed lines, between the TM-cyclen and the V_10_ anion.

Among the compounds where V_10_ acts as a ligand to coordination compounds, listed in Table S1, there are eight purely O_C_-bonded transition-metal complexes, three O_D_, six O_F_, five O_G_ and three mixed cases: one O_G_/O_F_, one O_G_/O_C_ and one O_D_/O_C_. In order to interpret the experimental findings concerning the preferred sites of protonation and to visualize nucleophilic molecular regions, the arrangement of electrostatic potential around the V_10_ surface has been done in a number of works.^23,24^ They revealed that O_B_ and O_C_ are the most basic accessible sites in [V_10_O_28_]^6–^ anion, making it the preferred site for fixation of inorganic complexes.^91^ In the literature, however, covalent interaction occurs primarily with the double-linked oxygen atoms of V_10_ (O_C_ and O_D_), followed by the terminal oxygens (O_F_ and O_G_), and there is no compound in which V_10_ binds through triple-linked oxygen (O_B_). The restriction to coordination of the triple-bridge oxygens has been thought to be caused by steric reasons.^25^

### Electrostatic Potential Surfaces

Electrostatic surface potential (ESP) calculations map spatially resolved electrostatic forces, which are derived from the quantum mechanical electron density and the positive contribution from the nuclear framework of a molecule. For inorganic complexes, ESP reveals regions of negative and positive potential that correspond to nucleophilic and electrophilic sites, respectively. These features help to rationalize reactive behavior, binding preferences and predict noncovalent interaction patterns through a three-dimensional representation. The ESP maps calculated for the optimized {TM­(cyclen)}^2+^ (TM = Ni­(II), Cu­(II) and Zn­(II)) moieties reveal distinct charge distribution, particularly in the region over the metal centers (Figure 5).

**Figure 5:**
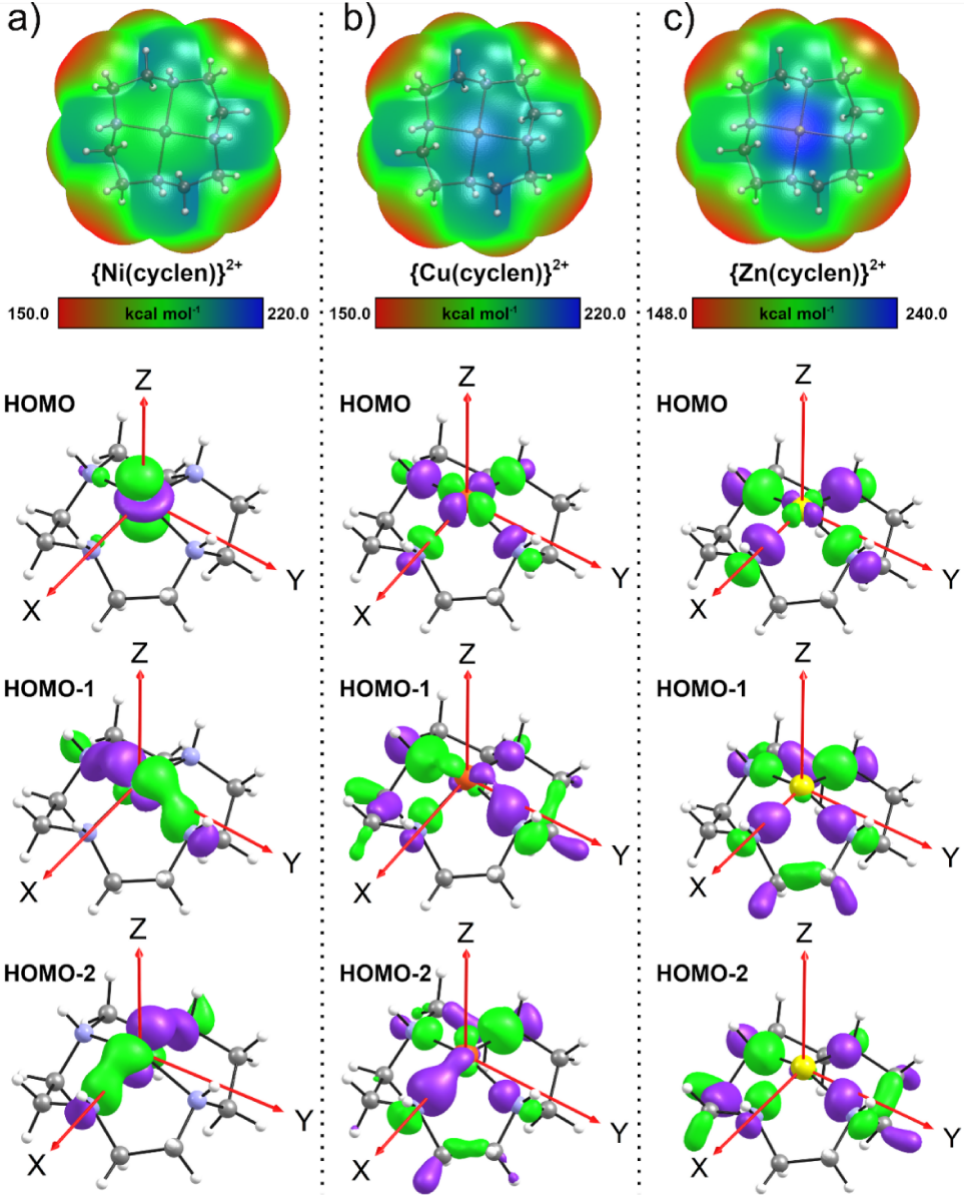
ESP map and frontier orbitals isosurfaces for (a) {Ni­(cyclen)}^2+^, (b) {Cu­(cyclen)}^2+^ and (c) {Zn­(cyclen)}^2+^.

For the {Ni­(cyclen)}^2+^complex, the maximum electrostatic potential is localized over the cyclen nitrogen atoms, reflecting the weak basicity of the metal ion. In contrast, both {Cu­(cyclen)}^2+^ and {Zn­(cyclen)}^2+^ exhibit π-holes over the molecular plane,^29^ with potential values of 233.1 and 197.8 kcal mol^–1^, favoring electrostatic interactions with the V_10_ anion. These directional long-range interactions may guide the association of the cations with V_10_ and are complemented by short-range forces, including hydrogen bonding and covalent interactions.

To better understand the nature of the π-hole in each {TM­(cyclen)}^2+^ moiety, their frontier orbital compositions were analyzed employing charge decomposition analysis (Figures 5 and S5– S7). For the 3d^8^ Ni­(II) system, the HOMO (Highest Occupied Molecular Orbital) displays a substantial contribution from the $d_{z^{2}}$ orbital (24%). The HOMO–1 and HOMO–2 show significant participation from the $d_{z^{2}}$ (≈26%), d_yz_ (27%), and d_xy_ (37%) orbitals, along with notable contributions from the p_x_ and p_y_ orbitals of the cyclen nitrogen atoms (≈35%). These orbital compositions are consistent with the expected electronic structure of a 3d^8^ cation in a square planar crystal field, where the antibonding $d_{x^{2}‐y^{2}}$ orbital remains unoccupied. This electronic configuration leads to strong σ-bonding between the ligand donors and the Ni­(II) center, favoring ligand-to-metal electron donation and thereby suppressing the formation of a π-hole at the metal center.

In contrast, the 3d^9^ Cu­(II) system exhibits a HOMO with substantial $d_{x^{2}‐y^{2}}$ character, as expected for a 3d^9^ cation. Partial occupation of this antibonding orbital weakens the σ bonds between the metal center and the ligands, leading to elongated M–N distances and reduced ligand-to-metal electron donation. This behavior is reflected in the nature of the lower-lying occupied orbitals (HOMO–2 and HOMO–3), which are dominated by the p_x_ and p_y_ orbitals of the cyclen nitrogen atoms (exceeding 75% contribution), indicating electron localization at the ligands. Although some metal participation is present, Cu­(II) is markedly less effective than Ni­(II) in accepting σ-donation, resulting in a net depletion of electron density at the metal center and the emergence of a π-hole in the ESP map.

Lastly, the 3d^10^ Zn­(II) system exhibits behavior analogous to that observed for Cu­(II). The HOMO shows a measurable $d_{x^{2}‐y^{2}}$ contribution (≈10%), consistent with occupation of an antibonding orbital. In this context, a 3d^10^ cation forms weaker σ-bonds than a 3d^9^ system, with even longer M–N distances, owing to the doubly occupied $d_{x^{2}‐y^{2}}$ orbital. The compositions of the HOMO–1 and HOMO–2 orbitals follow the same trend observed previously, with dominant contributions from the cyclen ligand. These results indicate that Zn­(II) has a very limited ability to accept σ-donation from the ligand, leading to substantial electron density depletion at the metal center and the formation of the most pronounced π-hole within the series.

ESP calculations were performed for the [V_10_O_28_]^6–^ and [H_2_V_10_O_28_]^4–^ anions to identify preferred coordination sites (Figure 6a,b). The nonprotonated [V_10_O_28_]^6–^ structure exhibits a symmetric ESP distribution, with triply bridging oxygen atoms (O_B_, Figure 1) showing the most negative electrostatic potential (−412 kcal mol^–1^), while terminal oxygen atoms (O_F_ and O_G_, −348 and −323 kcal mol^–1^ respectively) appear as electron-depleted regions. The double protonation to form [H_2_V_10_O_28_]^4–^ disrupts this symmetry, creating an asymmetric charge distribution across the V_10_ surface. Notably, one terminal oxygen (O_F_) becomes the second most basic site (−226 kcal mol^–1^) in the protonated form. These results are consistent with structural studies of V_10_-based minerals by Hawthorne et al.,^15^ demonstrating that protonation reduces the polyoxoanion’s Lewis basicity.

**Figure 6:**
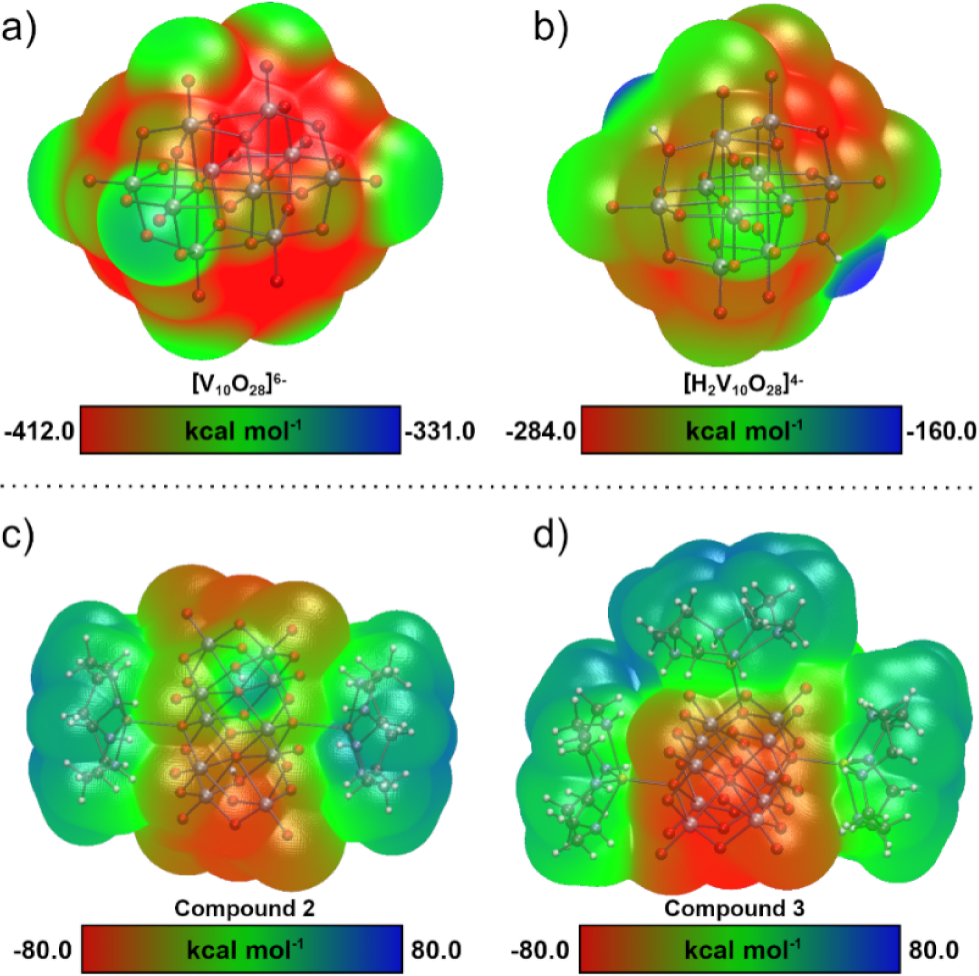
ESP map for (a) [V_10_O_28_]^6–^, (b) [H_2_V_10_O_28_]^4–^, (c) Compound **2** and (d) Compound **3**.

A comparative analysis of the ESP for **2** and **3** reveals lower surface negative charge in the Zn­(II) complex compared to the Cu­(II) analogue (Figure 6c,d). The ESP for **2** is elongated along the horizontal axis with a negative surface around the V_10_ moiety (−63 kcal mol^–1^) and a positive potential at the edges. For **3**, cyclen ligands are shielding the negative charge distribution of V_10_ (−76 kcal mol^–1^), covering three sides of the decavanadate with a neutral to positive potential surface (green to blue regions).

### Independent Gradient Model Analysis Based on Hirshfeld Partition (IGMH)

The nature of the noncovalent interactions and metal–ligand coordination was quantitatively evaluated using the Independent Gradient Model based on Hirshfeld partition (IGMH) methodology. This approach provides a sophisticated, quantum-mechanically rigorous advancement over the Noncovalent Interaction (NCI) index by enabling a fragment-partitionable analysis of chemical bonding. The core of the IGM method is the construction of a noninteracting promolecule, whose electron density (ρ__0__) is simply a superposition of the unperturbed, isolated atomic densities. The critical descriptor, δ_g_, is then defined as the difference between the normalized true electron density gradient of the interacting molecule (|∇ρ|) and the norm of the gradient of this noninteracting reference promolecule (|∇ρ__0__|), expressed as δ_g_ = |∇ρ| – |∇ρ__0__|.^92^ Therefore, the δ_g_ function isolates the perturbation in the electron density distribution caused by the formation of chemical interactions. In this context, IGMH refines the IGM approach by constructing its noninteracting reference state from Hirshfeld-partitioned deformed atoms, rather than spherical isolated atoms. This yields a new δ_g_ descriptor, that focus explicitly interfragment interactions discounting polarization effects, providing superior insight into intermolecular bonding.^93^

To interpret the electronic perturbations revealed by the IGMH analysis, the Laplacian of the electron density was employed, specifically the second eigenvalue (λ_2_) of its Hessian matrix. This parameter is a fundamental indicator of the local concentration (λ_2_ < 0) or depletion (λ_2_ > 0) of electron density. In this context, the product of the sign of the Laplacian by the electronic density is enough to characterize the electronic topology and distinguish between attractive and repulsive interactions providing a comprehensive frame of interatomic interactions.^94^

To characterize the interactions between {TM­(cyclen)}^2+^ cations and V_10_, the δ_g_ descriptor isosurfaces and corresponding scatter plots of δ_g_ versus sign­(λ_2_)­ρ were analyzed (Figure 7a,b). The product of the second Hessian eigenvalue sign, sign­(λ_2_), and the electron density (ρ) distinguishes attractive (sign­(λ_2_)­ρ < 0) from repulsive (sign­(λ_2_)­ρ > 0) interactions, visualized using a blue–green–red (BGR) color scale along the *x*-axis. Regions with significant δ_g_ and negative λ_2_ values indicate stabilized, shared electron density characteristic of bonding interactions (blue), whereas high δ_g_ coupled with positive λ_2_ values reflects repulsive forces, such as steric or electrostatic interactions (red). The ranges spanned from −0.05 to +0.04 for **2** and from −0.10 to +0.06 for **3**.^93^

**Figure 7:**
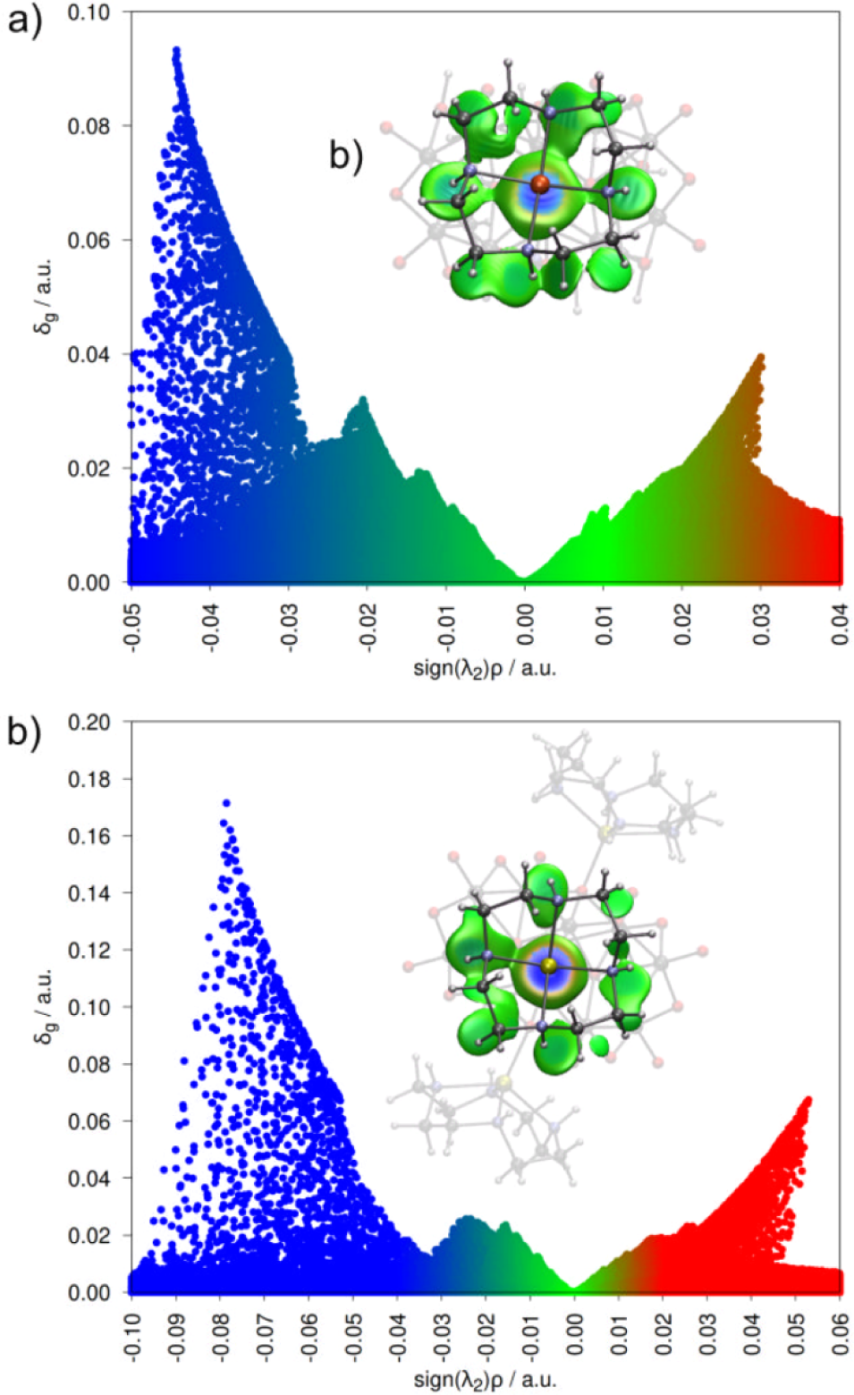
δ_g_ isosurface and δ_g_^inter^ scatter map as a function of *sign*(λ_2_ *)*ρ for: (a) diprotonated V_10_ and {Cu­(cyclen)}^2+^ fragments in **2**; (b) deprotonated V_10_ and {Zn­(cyclen)}^2+^ fragments in **3.** The δ_g_ isosurface is plotted at an isovalue of 0.003 and the scatter map uses a grid resolution of 0.01.

Comparative analysis highlights a distinct electronic interplay in **2** compared with **3**. The TM–V_10_ interaction in **3** shows twice the degree of electron sharing (δ_g_ ≈ 0.18) compared with the value for **2** (0.09), evidenced by the blue peaks in the scatter plots. Additionally, **3** displays a red peak at δ_g_ ≈ 0.06 and sign­(λ_2_)­ρ ≈ 0.05, indicative of a repulsive interaction around the Zn­(II) center and the O_B_ atom (reddish isosurface in the IGMH map). The higher δ_g_ in the Zn–O_B_ bond compared to the Cu–O_B_ suggests a more covalent character in **3**; however, its shorter bond length also introduces additional steric repulsion near the metal center. Finally, the presence of green regions in the δ_g_ isosurface plots for both compounds confirms extensive short-range noncovalent interactions, including hydrogen bonds between cyclen and V_10_, observed in the SC-XRD analysis.

### Intrinsic Bond Strength Index (IBSI)

To clarify the extent of the covalent contribution in both the Cu­(II) and Zn­(II) systems, the Intrinsic Bond Strength Index (IBSI) was calculated. The IBSI is a quantum-chemical descriptor used to quantify the absolute strength of a chemical bond between two atoms within a molecule.^35^ It is determined by integrating δ_g_ over the interatomic region within a cylindrical volume aligned with the bond vector of the atom pair. In essence, IBSI moves beyond simple topological analysis by providing a direct, quantitative measure of how much electron density is shared across the boundary between two atoms. This makes it particularly valuable for comparing bond strengths across different metal–ligand pairs or in varying molecular environments, as it offers a directly comparable numerical value. Values between 0 and 0.15 typically denoting weakly or noncovalent interactions, 0.15–0.60 being characteristic of coordination bonds and values above 0.60 indicating classical covalent bonds.^35^

For **2**, the Cu–O_B_ bond has a length of 2.251 Å and an IBSI value of 0.087, indicating a weakly covalent interaction, corresponding to roughly 60% of the strength of a typical coordinated bond. In this context, the interaction can be described as a semicoordination bond,^26,33,95^ primarily driven by electrostatic attraction between the π-hole on the {Cu­(cyclen)}^2+^ fragment and the V_10_ anion, which acts as a weak Lewis base. In contrast, **3** exhibits a Zn–O_B_ bond length of 1.987 Å and an IBSI of 0.169, falling within the range expected for a fully coordinated bond. The stronger electrostatic attraction between the {Zn­(cyclen)}^2+^ fragment and the anion allow for a closer TM–O_B_ proximity and enough orbital overlap to form a coordination bond.

The integrated theoretical analysis, including ESP, charge decomposition analysis, IBSI, and IGMH, reveals that TM–V_10_ bonding arises from a confluence of factors: orbital interactions, steric constraints imposed by the ligand, and the combined effects of noncovalent and intramolecular interactions. While the electrostatic potential of the V_10_ oxygen sites is frequently used to rationalize experimental results, their expected basicity follows the established nucleophilicity trend (O_B_ > O_C_ ≈ O_E_ > O_D_ > O_F_ ≈ O_G_). However, this ordering alone cannot fully explain the binding preferences of the TM complexes to V_10_. Therefore, identifying and considering other complementary interactions is essential for a comprehensive understanding of decavanadate coordination chemistry with other TM centers.

In order to place the interaction strengths observed in compounds **1**–**3** into context, additional IBSI calculations were performed on analogous mononuclear {TM­(cyclen)}^2+^ complexes [Ni­(cyclen)­(H_2_O)_2_]^2+^,^96^ [Cu­(cyclen)­(H_2_O)]2+^97^ and [Zn­(cyclen)­(H_2_O)]2+^98^ that do not contain decavanadate or other polyoxometalate anions. These reference systems provide a baseline for the intrinsic metal–ligand bonding within the cyclen coordination sphere, allowing a direct comparison with the interaction patterns observed in the present compounds. As shown in Figure 8, the IBSI values associated with the metal–nitrogen bonds in the reference complexes follow the order expected from the previously discussed δ-bonding rationalization for quasi-planar complexes containing Cu­(II) and Zn­(II), reflecting the relative δ-bonding strength, Cu­(II) > Zn­(II). In contrast, the reference Ni­(II) complex is octahedral and presents a coordination environment similar to that observed in compound **1**. This structural arrangement renders π-hole-driven interactions unlikely, and {Ni­(cyclen)­(H_2_O)_2_}^2+^ therefore behaves predominantly as an ion pair toward weakly coordinating anions such as decavanadate.

**Figure 8:**
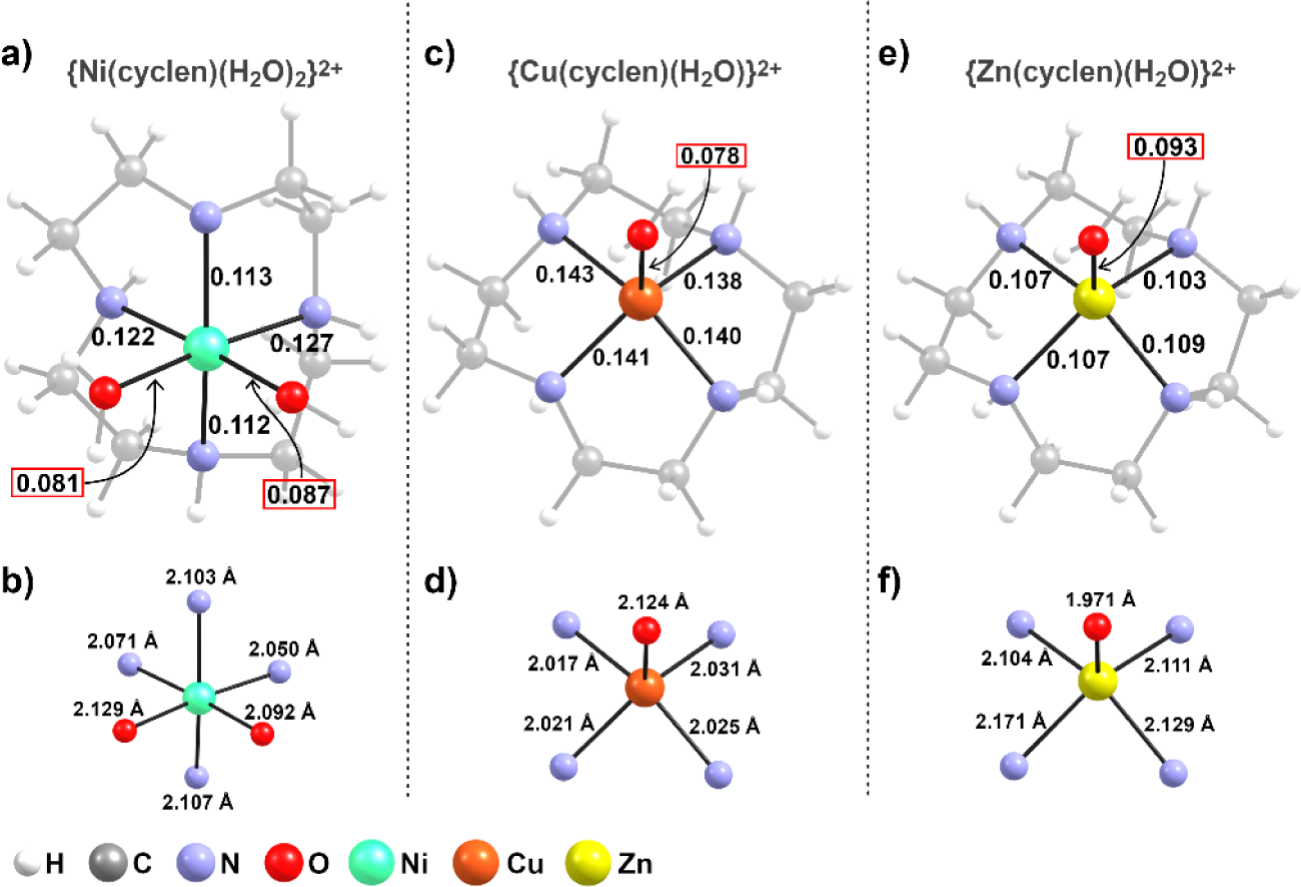
IBSI values and coordination geometries for literature {TM­(cyclen)}^2+^ complexes (TM = Ni, Cu, Zn) in the absence of decavanadate. Panels (a), (c), and (e) show the full coordination environments with selected IBSI values for metal–ligand interactions, while panels (b), (d), and (f) highlight the corresponding first coordination spheres with metal-donor bond distances. Hydrogen and carbon atoms are omitted for clarity.

The interactions involving coordinated water molecules exhibit consistently lower IBSI values than those observed for the TM-O_B_ contacts in compounds **2** and **3**, indicative of weaker and more labile bonding. In this context, the coordination of decavanadate to the {TM­(cyclen)}^2+^ fragment can be considered more favorable than coordination by water molecules, effectively driving the formation of hybrid materials.

### Solid-State Characterization of **1**–**3** in Bulk

The reaction between the orange solution of V_10_ and the blue solution of [Cu­(cyclen)­(H_2_O)]^2+^, both prepared *in situ*, immediately resulted in a green precipitate (**2**). The SEM images registered for **2** exhibit a heterogeneous distribution of crystal sizes, featuring mainly large prismatic crystals exceeding 10 μm along with approximately spherical agglomerates of crystals measuring less than 1 μm (Figure 9a,b and d). EDS analysis confirms that only V, O, C, Cu, and N atoms are present in the compound (Figures 9c and S8), consistent with the formula [{Cu­(cyclen)}_2_(H_2_V_10_O_28_)]·9H_2_O proposed by elemental analysis. The powder X-ray diffraction (PXRD) pattern of the green solid corresponds to that predicted by using the crystallographic data (Figure S9), indicating that the selected single crystal represents the whole product batch.

**Figure 9:**
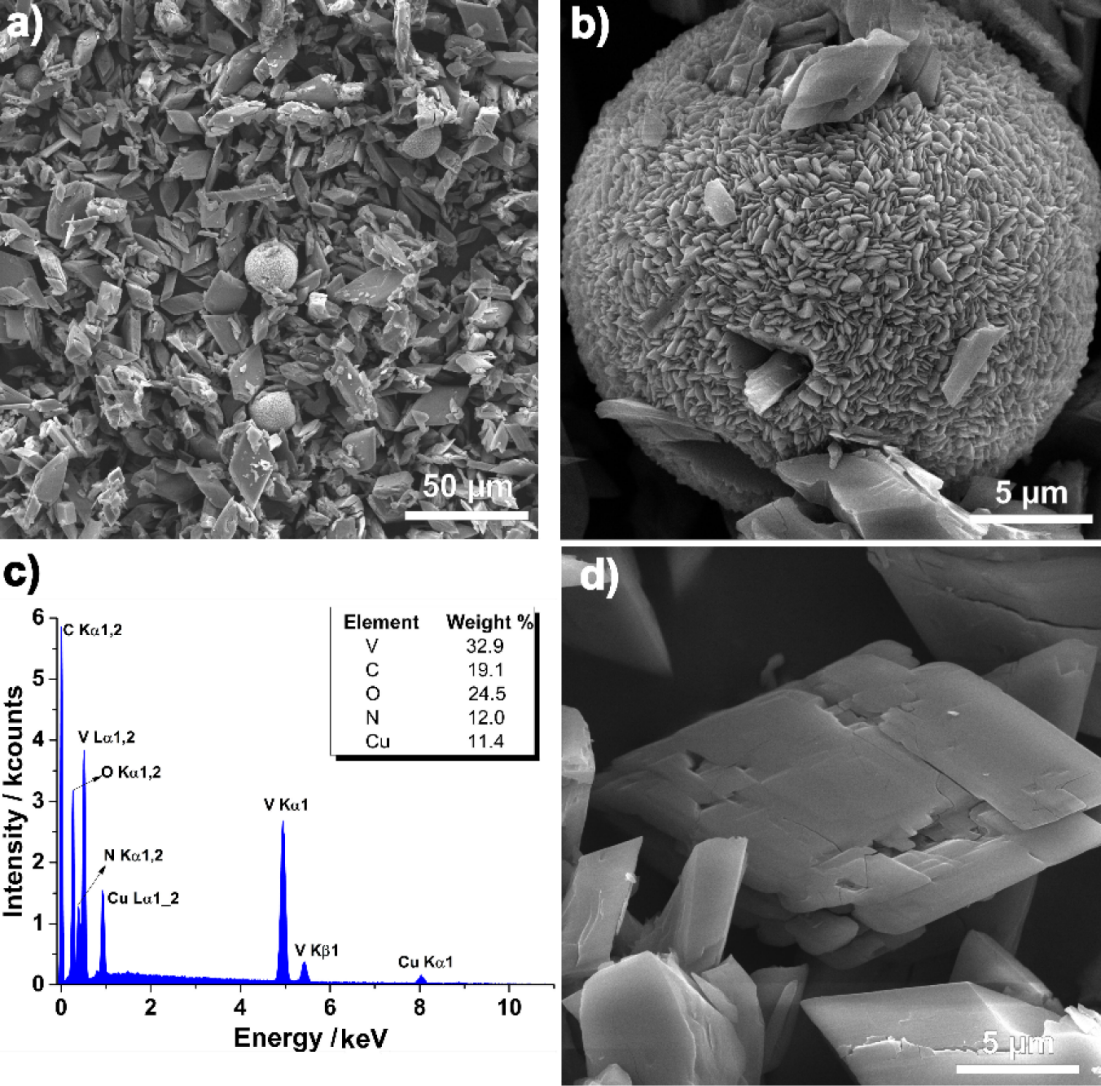
Scanning electron microscopy (SEM) images of **2** as obtained from the synthesis at magnification (a) 1.00 kx. Images (b) and (d) were taken from selected regions at magnification of 10.0 kx. (c) Energy-Dispersive X-ray Spectroscopy (EDS) spectrum displaying the partial element contents found for **2**.

In contrast, **1** and **3** were isolated as brown and orange crystals, respectively. The PXRD patterns of **1** and **2** showed good correspondence to those obtained from SC-XRD and were used as purity criterion along with elemental and EDS analyses (Figure S9). Even after the maceration of **1**, the resulting solid predominantly consisted of large crystalline fragments (>20 μm) covered by smaller crystallites (Figure 10a,b). After maceration of **2**, a narrower particle size distribution (0.5–2.5 μm) was found, leading to irregular crystalline grain boundaries (Figure 10c,d). Therefore, the increase in surface area may influence physicochemical properties such as reactivity and adsorption. Unlike **2**, solid **1** retained larger fragments, suggesting greater mechanical resistance. The maceration of **3**, in turn, rendered irregular particles of ca. 8–28 μm, without apparent crystallinity (Figure 10e,f), compatible with the low mechanical resistance of the crystals. The fragility of **3** had already become evident during the crystal selection for SC-XRD analysis, due to the loss of crystallinity under mechanical stress. The PXRD analysis of the macerated solid indicated a partial loss of crystallinity, as evidenced by the appearance of an amorphous halo. However, residual crystallinity was still observed, as evidenced by the diffraction peaks at 2θ = 8.7, 9.2, and 9.9°, which correspond to the most intense peaks in the diffraction pattern generated from the crystallographic data (Figure S9). Finally, EDS spectra of **1**–**3** (Figure S8) show the presence of the expected elements for the proposed formulations.

**Figure 10:**
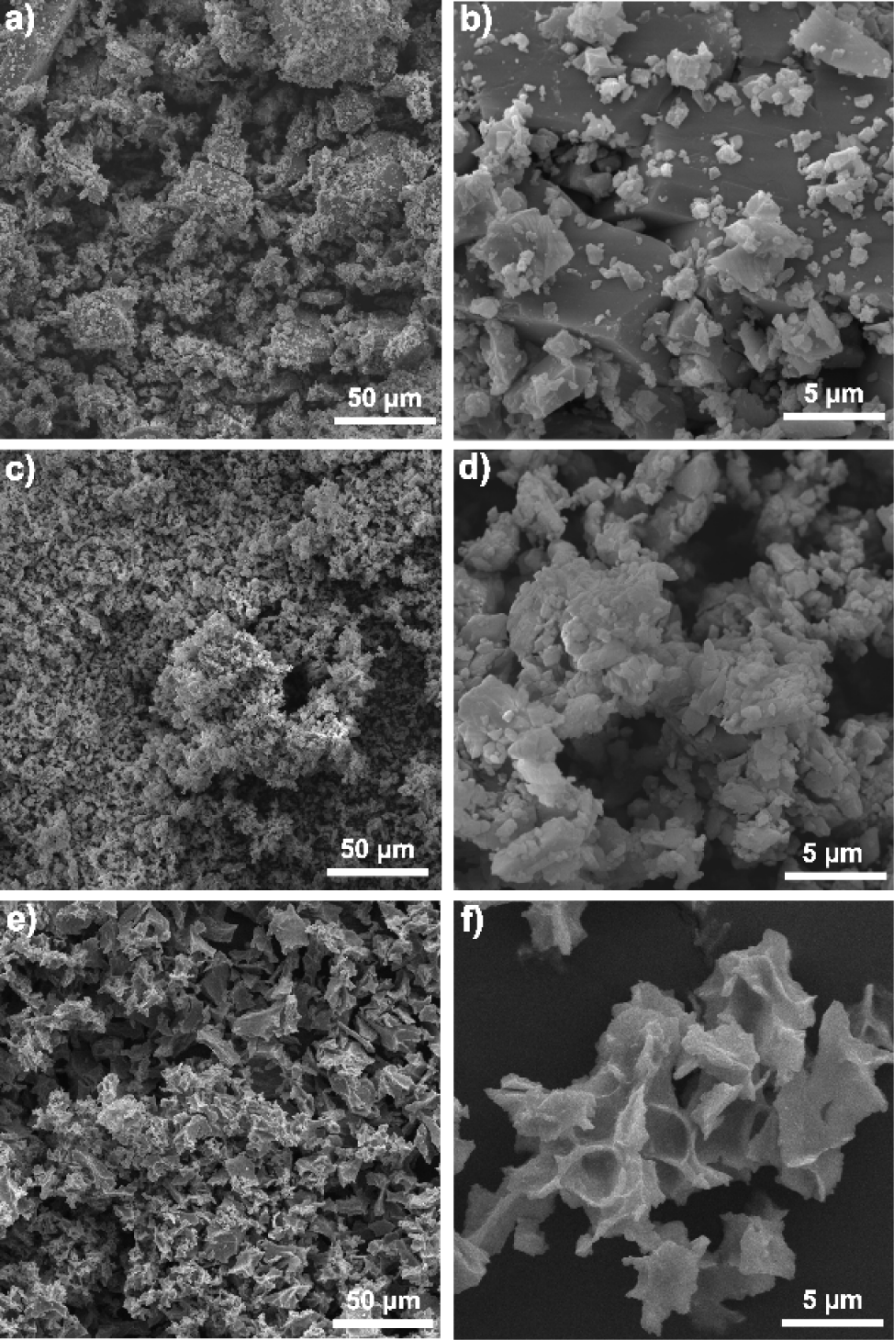
Scanning electron microscopy (SEM) images for macerated solids of **1** (a) and (b), **2** (c) and (d), and **3** (e) and (f).

The FTIR spectra of **1**, **2** and **3** exhibit characteristic bands of V_10_ at 1091–960 cm^–1^, 835–739 cm^–1^, 611–588 cm^–1^ and 447–432 cm^–1^ assigned to ν­(V=O), ν_*as*_(O–V–O), δ­(O–V–O) and ω­(O–V–O), respectively (Figure S10).^99^ The bands in the region above 3000 cm^–1^ are attributed to ν­(O–H) of the water molecules and ν­(N–H) of the macrocyclic ligand.^100^ Moreover, bands between 1473 and 1442 cm^–1^ are assigned to δ­(CH_2_) and ρ­(CH_2_).^101^ Further vibrations related to the cyclen ligand appear in the region between 2943 and 2863 cm^–1^, corresponding to weak ν­(C–H). The bending of water molecules is observed in the 1618–1600 cm^–1^ range.^102^

Complexes **1** and **3** were EPR-silent due to the diamagnetic properties of V­(V) and Zn­(II) (3d^0^ and 3d^10^ ions). Although Ni­(II) (3d^8^ ion, t_2_g^6^eg^2^ configuration) in an octahedral environment has unpaired electrons, the strong zero-field splitting hinders the detection of the spectrum.^103^ For **2**, the observed line in the EPR spectrum of the pulverized sample at 77 K is compatible with the presence of a Cu­(II) ion in a square-based pyramidal geometry (Figure S11).^104,105^ The EPR parameters [g_⊥_ (g_x_ = g_y_ = 2.085) and g_||_ (g_z_ = 2.124)] were calculated using DFT/B3LYP and *EasySpin* 4.0.0,^75^ reinforcing the anisotropy of the copper­(II) centers. Besides, the crystallographic data show Cu–N average equatorial bond lengths of 2.057(1) Å, shorter than the Cu–O axial bond length of 2.253(4) Å.

### Thermal Stability of **1**–**3**

The thermogravimetric analysis (TGA) and derivative thermogravimetry (DTG) results reveal similar decomposition patterns for **1–3**. The TGA data are summarized in Table 3 and are in good agreement with those obtained by elemental analysis. Product **1** exhibits two weight loss steps up to 238 °C, attributed to the release of four coordinated and four hydration water molecules (exp. 9.19% and calc. 9.20%) (Figure S12), as opposed to the two water molecules found in SC-XRD. This is followed by two superimposed weight loss steps up to 535 °C, assigned to the degradation of two cyclen molecules (exp. 22.19%, calc. 22.01%). Similar results were obtained in three TGA repetitions and are consistent with the elemental analysis; therefore, the formula [Ni­(cyclen)­(H_2_O)_2_]_2_[H_2_V_10_O_28_]·4H_2_O was used for adsorption studies. Products **2** and **3** also exhibited similar profiles, characterized by two initial weight loss steps, corresponding to the release of nine and four hydration water molecules, respectively. The decomposition of cyclen occurs in two steps for **2**, whereas for **3**, it is a multistep process in which the individual steps are overlapped. The final residues, observed at temperatures of 560 °C (**1**), 520 °C (**2**), and 550 °C (**3**), are likely composed of a mixture of vanadium pentoxide and other unidentified metal oxides.^9,106^

**Table 3:** Thermal Data for [Ni­(cyclen)­(H_2_O)_2_]_2_[H_2_V_10_O_28_]·4H_2_O (**1**), [{Cu­(cyclen)}_2_(H_2_V_10_O_28_)]·9H_2_O (**2**), [{Zn­(cyclen)}_3_(V_10_O_28_)]·4H_2_O (**3**)

			Weight loss (%)		
Samples	Steps	Temp. range (°C)	Experimental	Calculated	Possible moieties lost through decomposition
**1**	1–2	53–238	9.19	9.20	8 H_2_O
	3–4	238–535	22.19	22.01	2 cyclen
	Total^a^	-	**31.38**	**31.21**	-
**2**	1–2	30–240	11.57	11.30	9 H_2_O
	3–4	240–460	21.47	21.62	2 cyclen
	Total^a^	-	**33.04**	**32.92**	-
**3**	1–2	40–230	4.10	4.12	4 H_2_O
	3–4	230–520	28.91	29.66	3 cyclen
	Total^a^	-	**33.01**	**33.78**	-

a Total is related to the sum of the weight loss of water molecules and cyclen ligands.

### Adsorbent Properties of **1**–**3**

Bearing in mind the growing interest in POMs as organic-dye adsorbents, MB^+^ was employed as a model system to investigate the influence of structural features on the adsorption performance of these hybrid organic–inorganic materials. The adsorption studies of MB^+^ were conducted using the macerated solids of **1–3**. First, the influence of contact time was examined by mixing 10.0 mg of each POV with 100 mL of MB^+^ at 10 mg L^–1^ following the absorbance at 664 nm (Figures 11a,b and S13). Compound **2** was the most efficient, bleaching 84% of the MB^+^ solution, while **1** bleached 41% and **3**, only 3%, after 40 min. For **2**, the discoloration process is fast, achieving 71% in only 5 min (Figure 11c), reflecting a good interaction between the electrostatic surface of the POV and the cationic dye. The efficiency of **2** is similar to those reported for other decavanadate salts.^51–53^

**Figure 11:**
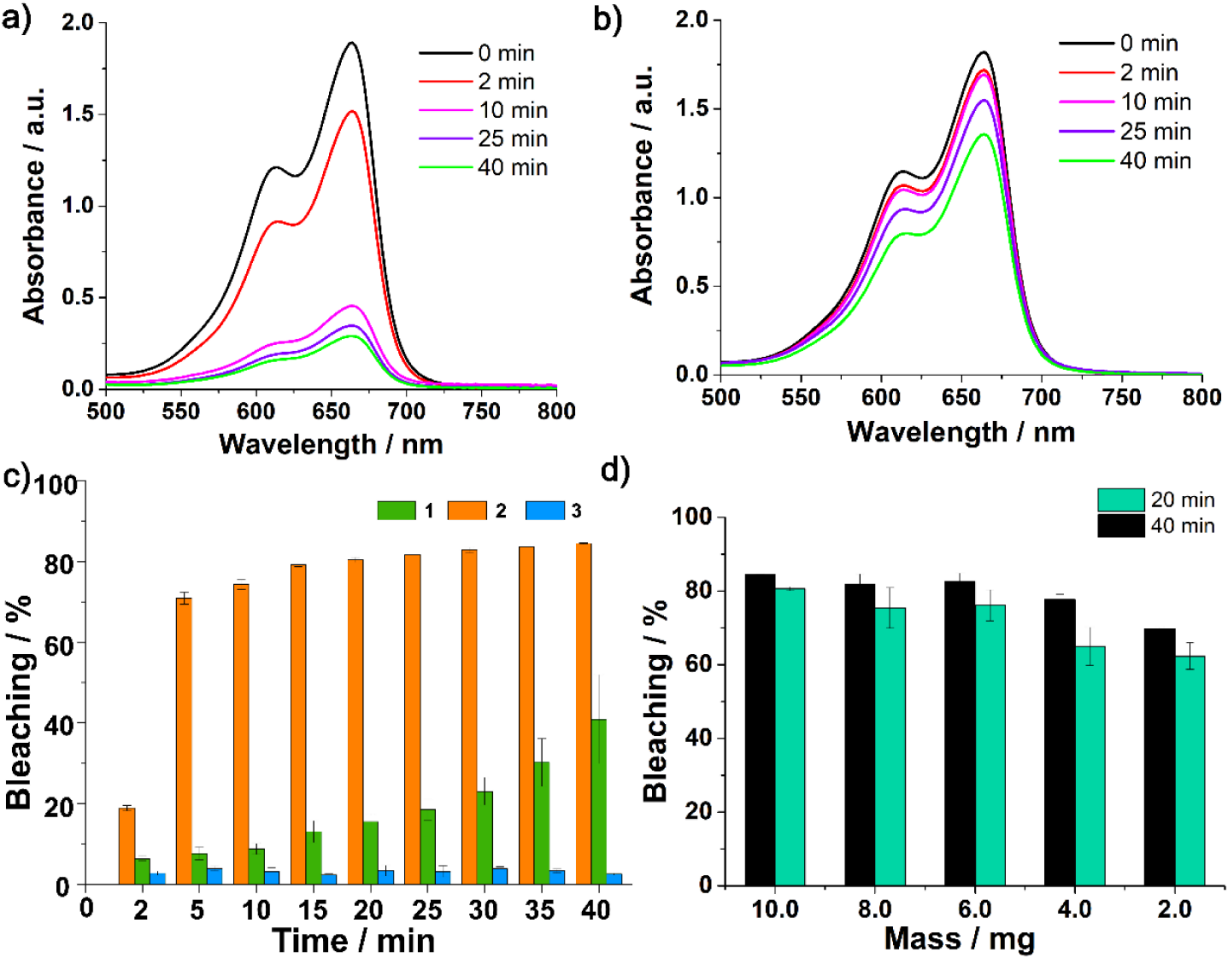
Electronic spectra of the supernatants of the bleaching reactions using (a) 10.0 mg of **2** and in (b) 10.0 mg of **1**, both added to a 10.0 mg L^–1^ aqueous solution of MB^+^. (c) Comparison of the activity of **1**, **2**, and **3** with standard deviation. (d) Activity of different amounts of **1** in 20 and 40 min in an aqueous solution of MB^+^ 10.0 mg L^–1^ with standard deviation.

Conversely, **1** proved to be less efficient than **2** due to its smaller surface area. In our previous work, it was demonstrated that water-soluble decavanadate salts render [MB]_4_[H_2_V_10_O_28_], which precipitates through a cation exchange process.^52^ However, for **1**, the observed discoloration appears to be associated with the additional breakage of particles during a prolonged stirring process, promoting an increased contact surface of the solid to interact with the dye. The bleaching efficiency of **3** remains at only 3% even after 4 h. The results mentioned above are in accordance with the arrangement of the complexes in the crystal lattices, the morphological studies and the charge distribution as seen in ESP maps. For **2**, the negative regions of V_10_, which interact with MB^+^ by electrostatic forces, are more exposed than in **3**.

Decreasing amounts of **2** with contact times of 20 and 40 min also showed good efficiency for the removal of MB^+^ (Figure 11d and Table S11). Attempts to add 1.0 mL of hydrogen peroxide did not result in a significant improvement of the bleaching effect (Figure S13), suggesting that the copper­(II) centers in **2** are not promoters of Fenton-like reactions. The characteristic bands of the reduced form of MB^+^, leuco-methylene blue, were not observed at 314 and 256 nm in the absorption spectra of the reaction mixture supernatant.^107^

The ESI mass spectra of the supernatants from experiments carried out with **2** displayed peaks of *m*/*z* 284 (MB^+^, C_16_H_18_N_3_S^+^), *m*/*z* 270 (Azure B, C_15_H_16_N_3_S^+^) and 268 (C_15_H_14_N_3_S^+^, assigned to MB^+^ having lost a methyl group as a methane molecule), expected for MB^+^ solutions (Figure S14).^108^ The FTIR profile of the remaining solid from the discoloration reaction using **2** as adsorbent (Figure S15) is similar to that registered for the hybrid material with additional weak bands characteristic of MB^+^ cations. Specifically, the absorption bands at 1602, 1480, 1395, and 1356 cm^–1^ assigned to ν­(C=N), ν­(C=S^+^), δ­(C–H) and ν­(C–N), respectively, confirm the presence of MB^+^ cations in the solid.^109^

To gain more insight into the adsorption features of **2**, the textural properties of the sample were assessed through N_2_ physisorption, and the resulting isotherm is depicted in Figure S16. Complex **2** exhibits a type II adsorption isotherm, characteristic of nonporous or macroporous materials.^110^ The hysteresis loop observed at high relative pressures is similar to type H3, characteristic of nonrigid aggregates of plate-like particles or due to a pore network consisting of macropores that are not completely filled with pore condensate.^110^ The calculated BET specific surface area was 6 m^2^ g^–1^, which is the same value estimated for the external surface area. Additionally, the detected micropore volume was 0.000 cm^3^ g^–1^, indicating the absence of micropores and confirming that the total area calculated by the BET equation corresponds to the external surface area. The measured total pore volume was 0.01 cm^3^ g^–1^, supporting the fundamentally nonporous nature of **2**. Therefore, the textural analysis has provided valuable insights into the adsorption behavior of **2**, suggesting that MB^+^ adsorption most likely takes place on its external surface.

The differences in dye-bleaching efficiency achieved with **2** and **3** can be rationalized considering the asymmetries in electronegative surface charge distributions generated by their distinct hybrid architectures (Figure 12). For **2**, the decavanadate-cyclen structural assemblies expose extended regions of alternating positive and negative electrostatic potential on the particle surface, facilitating the adsorption of MB^+^ through strong electrostatic interactions and leading to efficient dye removal. On the other hand, particles of **3** have a more homogeneous and less polarized surface, in which the distribution of positive and negative charge is significantly attenuated. As a consequence, the electrostatic interaction with MB^+^ is markedly weaker, resulting in limited adsorption and a substantially lower bleaching efficiency. These observations highlight that subtle differences in the building units of hybrid polyoxometalate-based materials translate into pronounced variations in macroscopic adsorption performance.

**Figure 12:**
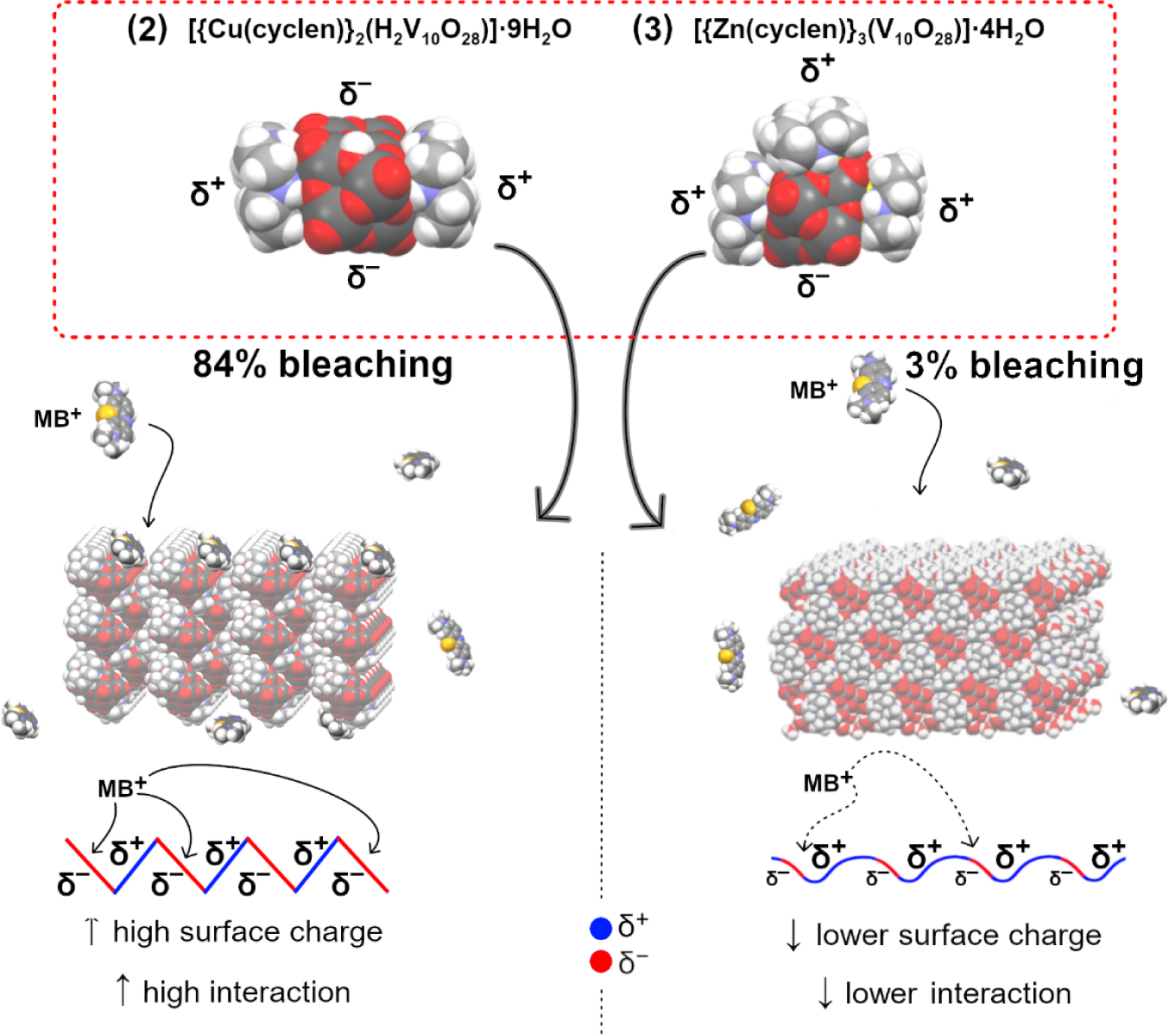
Schematic representation of the relationship between surface charge distribution and methylene blue (MB^+^) adsorption for compounds **2** and **3**. Compound **2** presents a highly polarized surface with alternating δ^+^/δ^–^ regions, promoting strong electrostatic interactions and high bleaching efficiency, whereas compound **3** displays a less polarized surface, resulting in weaker MB^+^ interactions and low bleaching efficiency. The solid-state arrangements for **2** and **3** are represented along the *c*-axis.

## Conclusion

Hybrid decavanadates with TM-complexes have yielded materials with diverse structural patterns and properties, suitable for applications in catalysis, energy storage, pharmaceuticals, and environmental chemistry. These materials are typically formed through self-assembly, leading to structures that vary from ionic pairs and discrete neutral compounds to one-dimensional chains or three-dimensional frameworks. In this work, we explored three distinct modes of interaction between the decavanadate anion and TM-cyclen complexes. These include forming ionic pairs (compound **1**), semicoordination bonds (compound **2**), and classical coordination bonds (compound **3**). In **2** and **3**, an unprecedented coordination mode of the decavanadate anion was observed via one of the most negatively charged oxygens (O_B_ in Figure 1), despite it being constrained by steric hindrance.

In this context, these TM-cyclen complexes proved to be a valuable model system for developing an approach that combines both experimental and theoretical analysis, not only to describe the strength of V_10_-TM bonds but also to better understand the driving forces behind the selection of the basic oxygen sites (O_B_, O_C_, O_D_, O_E_, O_F_, and O_G_) of V_10_ involved in the chemical bond. According to ESP analysis of the {TM­(cyclen)}^2+^ fragments, those cations exhibiting stronger π-holes above the molecular plane (Zn­(II) > Cu­(II)) are more prone to be coordinated by V_10_. ESP analysis also suggests that V_10_ can be seen as a highly negatively charged block that interacts with cationic complexes. IGMH analysis, on the other hand, showed that other complementary forces, including electrostatic and intramolecular noncovalent interactions between V_10_ and the cyclen ligand (N–H···O hydrogen bonds), are crucial to stabilizing the neutral hybrid structures. Furthermore, the O_B_-TM bond strength, quantified by IBSI values, revealed that Zn­(II) is coordinated to V_10_ in a conventional manner, whereas Cu­(II)–V_10_ interaction is significantly weaker, corresponding to a semicoordinate bond.

The three compounds were analyzed by multiple techniques to study their properties and structures. The arrangement of complexes within the crystal lattices reflects the negative charge distribution in each POV, which impacts the efficiency of bleaching MB^+^ aqueous solutions (**2** > **1** ≫ **3**). The bleaching effect was characterized as a superficial adsorption process, which does not involve cation exchange or Fenton-like reactions to dye degradation. Compound **2** is more effective than **1** due to its more electronegative surface potential, which enhances its affinity for the cationic dye, and because it has a higher negative surface area, despite lacking intrinsic porosity. Complex **3**, in turn, demonstrated limited adsorption capabilities, due to the position of the three {Zn­(cyclen)}^2+^ fragments that act as a barrier for electrostatic interactions between V_10_ and the MB^+^ cations.

To the best of our knowledge, this is the first time that ESP analysis has been combined with IGMH and IBSI to evaluate complex structural patterns commonly obtained in polyoxometalate chemistry. Finally, these findings advance the state of the art in polyoxometalate chemistry by integrating experimental and theoretical results to improve the understanding of the interactions governing the formation of hybrid species containing a second transition metal decorating the polyoxoanions, and their application in environmental chemistry.

## Supplementary Material



## Data Availability

The authors will make other raw data supporting this article’s conclusions available upon request.

## Notes

Complementary data tables, crystallographic data, infrared absorption spectra, powder diffraction patterns, SEM/EDS, thermogravimetric and DTG profiles, UV–vis absorption spectra, ESI-MS, N_2_ adsorption/desorption isotherm and DFT calculation tables and figures (PDF)

## Abbreviations

cyclen: 1,4,7,10-tetraazacyclododecane
POMs: Polyoxometalates
TM: Transition metal
DFT: Density Functional Theory
ESP: Electrostatic surface potential
IBSI: Intrinsic Bond Strength Index
IGM: Independent Gradient Model
IGMH: Independent Gradient Model based on Hirshfeld partitioning of the molecular density
NCI: Noncovalent Interaction
BGR: blue–green–red
MB^+^: Methylene blue
FTIR: Fourier-transformed infrared spectroscopy
TGA: Thermogravimetric analysis
PXRD: Powder X-ray diffraction
EPR: Electron paramagnetic resonance
SEM: Scanning Electron Microscopy
EDS: Energy Dispersive Spectroscopy
BET: Brunauer–Emmett–Teller
SC-XRD: Single-crystal X-ray diffraction
BVS: Bond valence sum
HOMO: Highest Occupied Molecular Orbital
