# Supplementary material for: Expanding the Coordination Chemistry of Decavanadate through π‑Hole Interactions with Transition-Metal Cyclen Complexes: Electronic Features and Dye Adsorption
Source: Inorg Chem. 2026 Mar 11;65(11):6266–83. doi: 10.1021/acs.inorgchem.6c00422 (PMC13014449; doi:10.1021/acs.inorgchem.6c00422)
Supplement: Supplementary file 1 [file ic6c00422_si_001.pdf]

Expanding the coordination chemistry of decavanadate through π-hole interactions with transition-metal cyclen complexes: electronic features and dye adsorption

Heloísa de Souza Camilo,[#] Lucas Gian Fachini,[#] Lorena Moreira Braga,[#] Gabriel Barros Baptistella,[#] Juliana Morais Missina,[#] Grazielli da Rocha,[#] Francine Bertella,[#] Patrizia Rossi,[&] Paola Paoli,[&] Eduardo Lemos de Sá,[#] Giovana Gioppo Nunes[#]

[#] *Departamento de Química, Universidade Federal do Paraná, Curitiba-PR, Brazil.*

[&] *Dipartimento di Ingegneria Industriale, Università degli Studi di Firenze, Firenze, Italy*

Corresponding author: Giovana Gioppo Nunes, e-mail: nunesgg@ufpr.br

SUPPORTING INFORMATION

Table S1. Coordination modes of $\{H_xV_{10}O_{28}\}^{(6-x)-}$ ions as ligands, where x = 2 to 4

Compound	Type of O atom coordinated	Type of O atom protonated	Geometry in heteroatom complex	Ref.
$[Cu(\mathit{1\text{-}pim})_4](H\mathit{pim})_4[V_{10}O_{28}]$	O_G	-	oh	1
$\{[(Cu\mathit{L}^{1})_{0.5}(H_2L)_{1.5}][H_2V_{10}O_{28}]\cdot 6H_2O\}_n$	O_F	O_B	oh	2
$(NH_4)_2[Cu_2(NH_3CH_2CH_2COO)_4(V_{10}O_{28})]\cdot 10H_2O$	O_G	-	tbp	3
$[Cu(cyclam)][\{Cu(cyclam)\}_2(V_{10}O_{28})]\cdot 10H_2O$	O_G, O_F	-	oh	4
$\{Cu(\mathit{pz})_4\}[\{Cu(\mathit{pz})_3\}_2V_{10}O_{28}]$	O_G, O_C	-	oh	5
$[Cu^{I}(H_x\text{dafone})_2]_3V^{IV}V_9{}^{V}O_{28}\cdot 5.25H_2O$ (x = 2/3)	O_D	-	oh	6
$[Cu^{I}(\text{dafone})_2]_2[Cu(OH_2)_5]_2[V_{10}O_{28}]\cdot 6.5H_2O$	O_G	-	oh	6
$[\{Cu(\mathit{en})_2\}_3(V_{10}O_{28})]\cdot 6H_2O$	O_D, O_C	-	sqp	7
$(H_3O)_2[\{Cu(en)_2(H_2O)\}_2V_{10}O_{28}]\cdot 3H_2O$	O_C	-	oh	7
$[Cu(\mathit{bpy})_2]_2[H_2V_{10}O_{28}]\cdot bpy\cdot H_2O$	O_C	$O_\#$	tbp	8
$[\{Cu(2\text{-}\mathit{amp})_2(OH_2)\}_2H_2V_{10}O_{28}]\cdot 4H_2O$	O_D	O_C	tbp	9
$(2\text{-}\mathit{hepH})_2[\{Cu(OH_2)_2(O,N\text{-}2\text{-}\mathit{hep})\}_2V_{10}O_{28}]\cdot 6H_2O$	O_C	-	oh	9
$(H\mathit{pz})_2[\{Cu(\mathit{pz})_4\}_2V_{10}O_{28}]\cdot 2H_2O$	O_G	-	oh	5
$[Cu(OH_2O)(\mathit{en})_2]_2[H_2V_{10}O_{28}]\cdot 12H_2O$	O_C	O_B	oh	10
$(\mathit{2\text{-}hepH})(NH_4)[\{Cu(H_2O)_2(2\text{-}hep)\}_2V_{10}O_{28}]\cdot 4H_2O$	O_C	-	tbp	9
$[Cu(en)_2V_{10}O_{28}][Cu(en)_2(H_2O)]2\cdot 2H_3BO_3\cdot 2H_2O$	O_E		sqp	11
$[Ni(1\text{-}\mathit{mim})_4(H_2O)_2][Ni(H_2O)_5]_2V_{10}O_{28}\cdot 5.5H_2O$	O_F	-	oh	12
$[Ni(1\text{-}\mathit{eim})(H_2O)_4]_2(V_{10}O_{28})(1\text{-}\mathit{eimH})_2\cdot 2H_2O$	O_F	-	oh	13
$[(CH_3)_4N]_2[Ni(H_2O)_5]_2V_{10}O_{28}\cdot 6H_2O$	O_F	-	oh	14
$.[Zn(\mathit{en})_2]_3[V_{10}O_{28}]\cdot 5H_2O$	O_C	-	oh and sqp	15
$.[Zn(H_2O)_6][Zn_2(btb)_2V_{10}O_{28}(H_2O)_6]\cdot 4H_2O$	O_G	-	oh	16
$[(CH_3)_4N]_2[Zn(H_2O)_5]_2V_{10}O_{28}\cdot 5H_2O$	O_F		oh	14
$.[Zn_2(H_2O)_{14}(V_{10}O_{28})]\cdot H_2ppz$	O_D	-	oh and sqp	17
$.[Zn(im)_2(dmf)_2]_2[H_2V_{10}O_{28}]\cdot im\cdot dmf$	O_C	O_B	tbp	18
$.\{[Zn_3(trz)_3(H_2O)_4(dmf)]_2[V_{10}O_{28}]\cdot 4H_2O\}_n$	O_C	-	tbp	18
$[HMTAH]_2[\{Zn(H_2O)_4\}_2\{V_{10}O_{28}\}]\cdot 2H_2O$	O_F,O_G		oh	19
$(HNAM)_2\{[Co(H_2O)_3(nam)_2]_2[\mu\text{-}V_{10}O_{28}]\}\cdot 6H_2O$	O_F	-	oh	20
$\{[Co(H_2O)_4]_2[Co(H_2O)_2(\mu\text{-}pza][\mu\text{-}V_{10}O_{28}]\}\cdot 4H_2O$	O_F	-	oh	20
$[(CH_3)_4N]_2[Co(H_2O)_5]_2V_{10}O_{28}\cdot 6H_2O$	O_F	-	oh	14
$(2\text{-}hepH)_2[\{Co(H_2O)_5\}_2V_{10}O_{28}]_4H_2O$	O_F	-	-	21
$[Ag_2(1\text{-}\mathit{eim})_4]_2[Ag(1\text{-}\mathit{eim})_2]_3\cdot 2Ag(1\text{-}\mathit{eim})_2\cdot 3(1\text{-}H\mathit{eim})[V_{10}O_{28}]_2$	O_F, O_C	-	$\{AgN_2O_2\}$ $\{AgN_2O\}$, sq and linear	22
$[Ag(1\text{-}\mathit{pim})_2]_3[HV_{10}O_{28}]\cdot {}_2Ag(1\text{-}\mathit{pim})_2\cdot 2H_2O$	O_G	-	oh, AgN_2O_2	22
$[Ag(btx)]_4[H_2V_{10}O_{28}]\cdot 2H_2O$	O_C	O_C	Linear	23
$\{(Ag(CH_3CN)_3)_3[H_3V_{10}O_{28}].\ CH_3CN\}_2$	O_C	-	td	24
$Ag_3(dmso)_6[Ag_1(dmso)_3][H_2V_{10}O_{28}].1dmso\}n$	O_C,O_E,O_G	-	Linear	25
$Ag_3(dmso)_6[Ag_1(dmso)_2][H_2V_{10}O_{28}].2dmso\}n$	O_C,O_E,O_G	-	Linear	25
$[Mn(mim)_4]_2[H_2V_{10}O_{28}]$	O_G, O_F	$O_\#$	oh	26
$[(CH_3)_4N]_2[V_{10}O_{28}\{Mn(H_2O)_5\}_2]\cdot 5H_2O$	O_F	-	oh	27
$[NH_3C(CH_2OH)_3]_2[V_{10}O_{28}\{Mn(H_2O)_5\}_2\cdot 2H_2O$	O_F	-	oh	27
$[(CH_3)_4N]_2[Mn(H_2O)_5]_2V_{10}O_{28}\cdot 4H_2O$	O_F	-	oh	14

Abbreviations: 1-pim = 1-isopropylimizadole, L^1= 5,5,7,12,12,14-hexamethyl-1,4,8,11-tetracyclotetradecane, cyclam= 1,4,8,11-tetraazacyclotetradecane, pz = pyrazol, dafone = 4,5-diazafluoren-9-one, en = ethylenediamine, bpy = bipyridine, 2-amp = 2-(aminemethyl)pyridine, 2-hepH = 2-hydroxyethylpyridine, mim = 1-methylimidazole, eim=1-ethylimidazole, btx = 1,4-bis(triazol-1-methyl)benzene, btb = 1,4-bis(1,2,4-triazol-1-y1)butane, dmf = N,N'-dimethylammonium, im = imidazole, ppz = pipeazine, trz = 1,2,4-triazole, nam = nicotinamide, pza = pyrazinamide, 1-pim = 1-propylimizadole, NMe_4 = tetramethylammonium. Abbreviations at geometry of MT^{II} section corresponds to sqp = square pyramidal, oh = octahedral, tbp = trigonal bipyramid, td = tetrahedral. $O_\#$ = undefined protonated oxygen

Table S2. Bond lengths (Å) and angles (°) for the nickel coordination sphere in $[Ni(cyclen)(H_2O)_2]_2[H_2V_{10}O_{28}]\cdot 2H_2O$ (**1**) with estimated standard deviations in parentheses.

Bond	Length (Å)
Ni-N(1)	2.107(2)
Ni-N(2)	2.081(2)
Ni-N(3)	2.111(2)
Ni-N(4)	2.048 (2)
Ni-O(1W)	2.128(2)
Ni-O(2W)	2.118(2)

Bond	Angle(°)
N(4)-Ni-N(2)	99.71(7)
N(4)-Ni-N(1)	85.19(8)
N(2)-Ni-N(1)	82.21(8)
N(4)-Ni-N(3)	85.02(8)
N(2)-Ni-N(3)	81.33(8)
N(1)-Ni-N(3)	159.12(8)
N(4)-Ni-O(1W)	87.78(8)
N(2)-Ni-O(1W)	172.23(7)
N(1)-Ni-O(1W)	100.60(9)
N(3)-Ni-O(1W)	97.40(9)
N(4)-Ni-O(2W)	168.93(7)
N(2)-Ni-O(2W)	90.92(7)
N(1)-Ni-O(2W)	93.25(7)
N(3)-Ni-O(2W)	99.75(8)
O(1W) -Ni-O(2W)	81.72(8)

Table S3. Hydrogen bonds observed in the crystal structure of $[Ni(cyclen)(H_2O)_2]_2[H_2V_{10}O_{28}]\cdot 2H_2O$ (**1**) . Distances are expressed in Angstroms (Å) and angles in degrees (°)

**D-H···A**	**d(D-H)** Å	**d(H···A)** Å	**d(D···A)** Å	**<(DHA)** °
		Compound **1**		
N(1)-H(1)...O(6)#1	0.83(0.03)	2.57(0.03)	3.186(0.003)	132(2)
N(1)-H(1)...O(7)#1	0.83(0.03)	2.61 (0.03)	3.220(0.002)	132(2)
N(2)-H(2)...O(5)#2	0.83(0.03)	2.45(0.03)	3.279(0.002)	169(3)
N(3)-H(3)...O(6)#3	0.76(0.03)	2.52 (0.03)	3.206(0.003	149(3)
N(3)-H(3)...O(10)	0.76(0.03)	2.49 (0.03)	3.118(0.003)	141(3)
N(4)-H(4)...O(2)#4	0.88(0.03)	2.30(0.03)	3.098(0.003)	151(3)
O(3)-H(3O3)...O(8)#1	0.65(0.03)	2.10(0.03)	2.745(0.002)	169(3)
O(1W)-H(1WA)...O(13)#3	0.82(0.02)	2.30(0.02)	3.117(0.003)	176(2)
O(1W)-H(1WB)...O(3W)	0.82(0.02)	2.16(0.02)	2.775(0.003)	132(2)
O(2W)-H(2WA)...O(10)	0.82(0.01)	2.48(0.01)	3.095(0.002)	133(1)
O(2W)-H(2WA)...O(9)	0.82(0.01)	2.19(0.02)	2.953(0.002)	154 (2)
O(2W)-H(2WB)...O(8)#1	0.82(0.01)	2.35(0.03)	2.994(0.003)	136(1)
O(2W)-H(2WB)...O(7)#1	0.82(0.01)	2.53(0.01)	3.295(0.002)	156.5(0.9)
O(3W)-H(3WA)...O(2)#1	0.82(0.01)	2.17 (0.01)	2.956(0.003)	161.6(0.9)
O(3W)-H(3WB)...O(12)#3	0.82(0.03)	2.18(0.02)	2.941(0.003)	154(2)

Symmetry transformations used to generate equivalent atoms:
#1: 1-x, 1-y, 1-z; #2: 1.5-x, 0.5+y, 1.5-z; #3: 1-x, -y, 1-z;; #4: -0.5+x, 0.5-y, 0.5+z

Table S4. Selected bond lengths (å) and angles (°) for $[\{Cu(cyclen)\}_2(H_2V_{10}O_{28})]^{\cdot}9H_2O$ (**2**) with estimated standard deviations in parentheses

Bond	Length (Å)
Cu(1)-N(1)	2.008(4)
Cu(1)-N(2)	2.018(4)
Cu(1)-N(3)	2.015(5)
Cu(1)-N(4)	2.022(4)
Cu(1)-O(13)	2.253(4)

Bond	Angle (°)
N(1)-Cu(1)-N(2)	85.93(2)
N(1)-Cu(1)-N(3)	149.8 (2)
N(1)-Cu(1)-N(4)	86.7(2)
N(2)-Cu(1)-N(3)	86.2(2)
N(2)-Cu(1)-N(4)	150.3(2)
N(3)-Cu(1)-N(4)	85.8(20)
N(1)-Cu(1)-O(13)	102.4(2)
N(2)-Cu(1)-O(13)	109.9(2)
N(3)-Cu(1)-O(13)	107.6(2)
N(4)-Cu(1)-O(13)	99.8(2)

Table S5. Hydrogen bonds observed in the crystal structure of $[\{Cu(cyclen)\}_2(H_2V_{10}O_{28})]^{\cdot}9H_2O$ (**2**). Distances are expressed in Angstroms (Å) and angles in degrees (°)

D-H···A	d(D-H) Å	d(H···A) Å	d(D···A) Å	<(DHA) °
N(1)-H(1N1)...O(9)#1	0.91(0.06)	2.03(0.06)	2.903(0.006)	162(5)
N(2)-H(1N2)...O(6)	0.82 (0.07)	2.70(0.06)	3.320(0.006)	133(5)
N(3)-H(1N3)...O(5)	0.75(0.06)	2.25(0.07)	2.915(0.006)	147(6)
N(4)-H(1N4)...O(8)#1	0.84(0.06)	2.40((0.07)	3.021(0.006)	131(5)
N(4)-H(1N4)...O(12)	0.84(0.06)	2.63(0.06)	3.266(0.006)	133 (5)

Symmetry transformations used to generate equivalent atoms:
#1: -x+1, -y+1, -z+1

Table S6. Selected bond lengths (Å) and angles (°) for [{Zn(cyclen)}$_3$($V_{10}O_{28}$)]·4H_2O (**3**) with estimated standard deviations in parentheses

**Bond**	**Length (Å)**	**Bond**	**Length (Å)**	**Bond**	**Length (Å)**
Zn(1A)-O(13A)	1.992(3)	Zn(1B)-O(13B)	1.973(3)	Zn(1C)-O(7B)	1.997(3)
Zn(1A)-N(1A)	2.142(5)	Zn(1B)-N(1B)	2.146(5)	Zn(1C)-N(1C)	2.175(5)
Zn(1A)-N(2A)	2.140(4)	Zn(1B)-N(2B)	2.122(4)	Zn(1C)-N(2C)	2.122(4)
Zn(1A)-N(3A)	2.158(4)	Zn(1B)-N(3B)	2.143(4)	Zn(1C)-N(3C)	2.129(4)
Zn(1A)-N(4A)	2.126(4)	Zn(1B)-N(4B)	2.124(4)	Zn(1C)-N(4C)	2.135(4)

**Bond**	**Angle (°)**	**Bond**	**Angle (°)**	**Bond**	**Angle (°)**
O(13A)-Zn(1A)-N(1A)	113.9(1)	O(13B)-Zn(1B)-N(1B)	111.5(2)	O(7B)-Zn(1C)-N(1C)	116.4(1)
O(13A)-Zn(1A)-N(2A)	108.1(1)	O(13B)-Zn(1B)-N(2B)	113.2(1)	O(7B)-Zn(1C)-N(2C)	106.7(1)
O(13A)-Zn(1A)-N(3A)	110.4(1)	O(13B)-Zn(1B)-N(3B)	112.2(1)	O(7B)-Zn(1C)-N(3C)	108.7(1)
O(13A)-Zn(1A)-N(4A)	116.8(1)	O(13B)-Zn(1B)-N(4B)	111.1(1)	O(7B)-Zn(1C)-N(4C)	116.5(1)
N(2A)-Zn(1A)-N(1A)	82.1(2)	N(1B)-Zn(1B)-N(3B)	136.2(2)	N(2C)-Zn(1C)-N(1C)	81.3(2)
N(2A)-Zn(1A)-N(3A)	81.4(2)	N(2B)-Zn(1B)-N(1B)	82.0(2)	N(2C)-Zn(1C)-N(4C)	136.7(2)
N(3A)-Zn(1A)-N(1A)	135.6(2)	N(2B)-Zn(1B)-N(3B)	81.9(2)	N(3C)-Zn(1C)-N(1C)	134.8(2)
N(3A)-Zn(1A)-N(4A)	81.9(2)	N(4B)-Zn(1B)-N(1B)	81.8(2)	N(3C)-Zn(1C)-N(4C)	81.9(2)
N(4A)-Zn(1A)-N(1A)	81.442)	N(4B)-Zn(1B)-N(2B)	135.8(2)	N(3C)-Zn(1C)-N(2C)	83.1(2)
N(4A)-Zn(1A)-N(2A)	135.0(2)	N(4B)-Zn(1B)-N(3B)	82.0(2)	N(4C)-Zn(1C)-N(1C)	81.1(2)

Table S7. Hydrogen bonds observed in the crystal structure of [{Zn(cyclen)}$_3$($V_{10}O_{28}$)]·4H_2O (**3**). Distances are expressed in Angstroms (Å) and angles in degrees (°)

**D-H···A**	**d(D-H)** Å	**d(H···A)** Å	**d(D···A)** Å	**<(DHA)** °
N(1A)–H(1A)···O(12A)	0.70(0.07)	2.54(0.07)	3.108(0.006)	139(8))
N(2A)–H(2A)···O(5A)	0.93(0.06)	2.04(0.06)	2.879(0.005)	149(5)
N(3A)–H(3A)···O(6A)	0.80(0.07)	2.37(0.06)	3.008(0.006)	138(6)
N(4A)–H(4A)···O(9B)	0.79(0.08)	2.33(0.07)	2.900(0.005)	130(6)
N(2B)–H(2B)···O(9A)	0.86(0.06)	2.10(0.06)	2.870(0.006)	149(6)
N(4B)–H(4B)···O(5B)	0.71(0.06)	2.31(0.06)	2.935(0.005)	148(6)
N(1C)–H(1C)···O(2B)	0.86(0.07)	2.38(0.08)	3.004(0.006)	130(6)
N(3C)–H(3C)···O(12A)	0.88(0.04)	2.20(0.05)	2.978(0.005)	147(5)
N(4C)–H(4C)···O(3B)	0.89(0.08)	2.22(0.07)	2.890(0.005)	132(6)
N(1A)–H(1A)···O(3W3)#1	0.70(0.07)	2.50(0.07)	3.015(0.006)	132(8)
N(3A)–H(3A)···O(4W4)	0.80(0.07)	2.58(0.07)	3.268(0.006)	145(6)
O(1W1)–H(1W1)···O(3A)#2	0.85(0.04)	1.96(0.04)	2.799(0.005)	171(4)
O(1W1)–H(2W1)···O(1B)	0.84(0.04)	1.93 (0.04)	2.768(0.005)	177(4)
O(2W2)–H(1W2)···O(12A)#3	0.85(0.05)	2.11(0.05)	2.908(0.005)	157(5)
O(2W2)–H(2W2)···O(1W1)	0.85(0.04)	1.89(0.03)	2.719(0.006)	167(6)
O(3W3)–H(1W3)···O(2W2)	0.84(0.04)	1.97(0.05)	2.792(0.006)	165(4)
O(3W3)–H(2W3)···O(4W4)#2	0.84(0.04)	2.08(0.05)	2.886(0.006)	161(5)
O(4W4)–H(2W4)···O(10A)#4	0.84(0.03)	2.05(0.02)	2.874(0.006)	169(5)

Symmetry transformations used to generate equivalent atoms:
#1: x,1-y,-0.5+z; #2: 0.5+x,0.5-y,0.5+z; #3: x,1-y,0.5+z; #4: x,-1+y,+z

Table S8. Bond valence calculations for oxygen atoms in $[H_2V_{10}O_{28}]^{4-}$ for **1**

O atom	V atom	V-O length	$s = (r/1.791)^{-5.1}$	$\sum s$
O4	V1	2.0229(15)	0.5374	1.912
	V4	1.6828(14)	1.3741	
O9	V3	2.0764(15)	0.4704	1.836
	V4	1.9073(13)	0.7255	
	V5	1.9549(13)	0.6398	
O2	V1	1.8521(14)	0.8428	1.758
	V2	1.8222(14)	0.9157	
O7	V2	1.9480(15)	0.6515	1.812
	V3	1.7396(15)	0.4704	
O13	V2	1.8369(15)	0.8789	1.787
	V5	1.8253(15)	0.9078	
O5	V1	1.8023(15)	0.9684	1.761
	V5	1.8747(14)	0.7922	
O3	V1	1.9966(15)	0.5745	1.209
	V3	1.9581(15)	0.6345	
O10	V2	2.0358(15)	0.5202	1.830
	V4	1.6988(15)	1.3094	
O11	V3	1.9184(13)	0.7044	1.951
	V4	1.9406(14)	0.6642	
	V5	1.9914(15)	0.5822	

Table S9. Bond valence calculations for oxygen atoms in $[H_2V_{10}O_{28}]^{4-}$ for **2**

O atom	V atom	V-O length	$s = (r/1.791)^{-5.1}$	$\sum s$
O2	V1	1.977(38)	0.6040	1.894
	V2	1.7038(37)	1.289	
O9	V3	1.8328(35)	0.8889	1.702
	V4	1.8648(40)	0.8127	
O3	V1	1.8822(43)	0.7752	1.613
	V3	1.8548(42)	0.8381	
O4	V1	1.8618(38)	0.8217	1.839
	V4	1.7855(39)	1.017	
O5	V1	1.8938(39)	0.7515	1.714
	V5	1.8040(38)	0.9629	
O11	V4	1.9408(40)	0.6646	1.4082
	V5	1.8974(37)	0.7436	
O6	V2	1.6783(36)	1.3947	1.885
	V4	2.0597(37)	0.4906	
O13	V2	1.9896(38)	0.5841	1.750
	V3	2.0033(36)	0.5646	
	V5	1.9790(35)	0.6009	
O7	V2	1.8858(40)	0.7677	1.950
	V3	1.9728(35)	0.6103	
	V5	1.9987(38)	0.5719	

Table S10. Tentative assignments for the infrared absorption spectra (cm^{-1}) of of **1, 2** and **3**[28]

Tentative assignments	**1**	**2**	**3**
ν_s(V=O)	1012, 960	997, 960	963
ν_{as}(O–V–O)	829, 748	835, 739	826, 743
δ(O–V–O)	611	601	588
ν (O–H)	3543	3568, 1637	3568
ν(N–H)	3323, 3233	3278,3224	3259
δ(N–H)	1473, 1454	1448, 1448	1442, 1227
ν(C–N)	1110, 1042	1123, 1080	1091
ν (C–H)	2943, 2883	2927, 2863	2927, 2863
ν (V–O)	447	432	433
ν_{as}(V–O–V)	829, 748, 611	835, 739, 601, 549	826, 743, 588

ν = stretching, ν_{as} = asymmetric stretching, ν_{sym} = symmetric stretching, and δ = in- or out-of-plane bending

Table S11. Discoloration values of MB solution at 10.0 mg L^{-1} bleached by **2** represented by arithmetic mean (%) ± standard deviation (SD) in the different reaction conditions

**Reaction condition**	**Reaction time (min)**	
*Load level of **2***	20 min	40 min
	**Bleaching (%) ± SD**	
2.0 mg	62.37 ± 3.6	69.75 ± 0.44
4.0 mg	64.99 ± 5.2	77.69 ± 1.4
6.0 mg	76.15 ± 4.2	82.54 ± 2.4
8.0 mg	75.44 ± 5.5	81.78 ± 2.8
10.0 mg	80.65 ± 0.53	84.39 ± 0,19
Presence of 1.0 mL H_2O_2		
10.0 mg of 2	30 min	80.68 ± 2.31

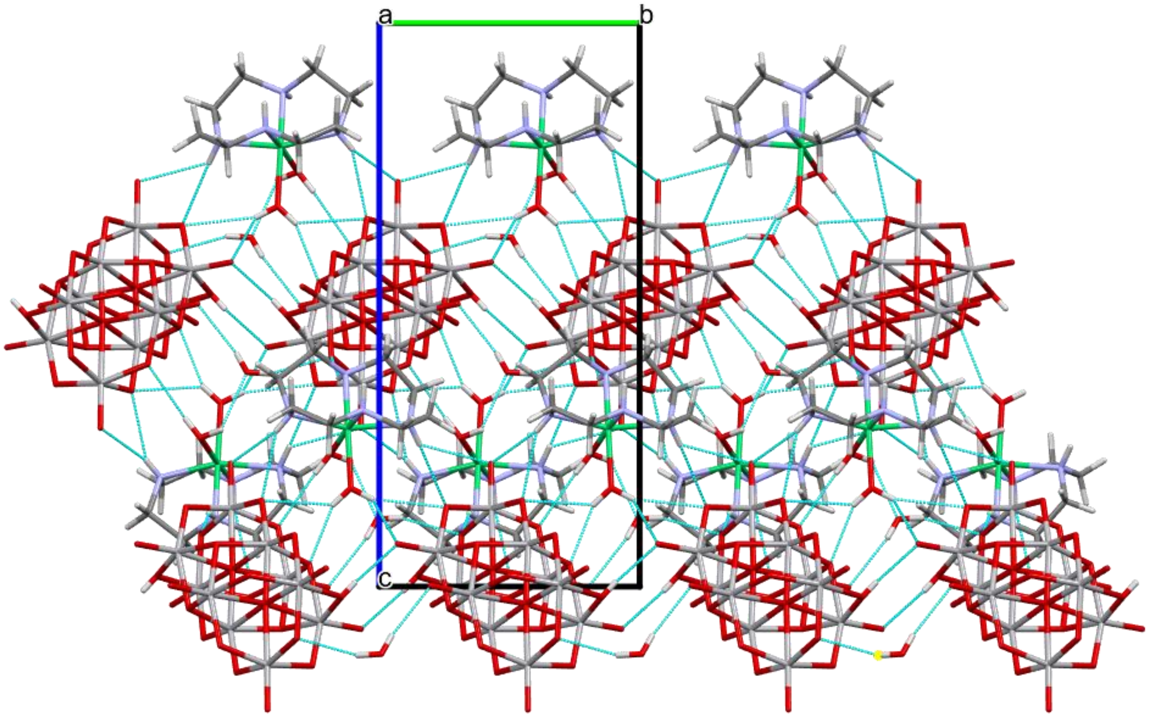

Figure S1. a) View along the a-axis direction of the crystal packing of $[Ni(OH_2)_2(cyclen)]_2[H_2V_{10}O_{28}]\cdot 2H_2O$ highlighting the alternating V_{10}-based chains and Ni-cyclen complexes.

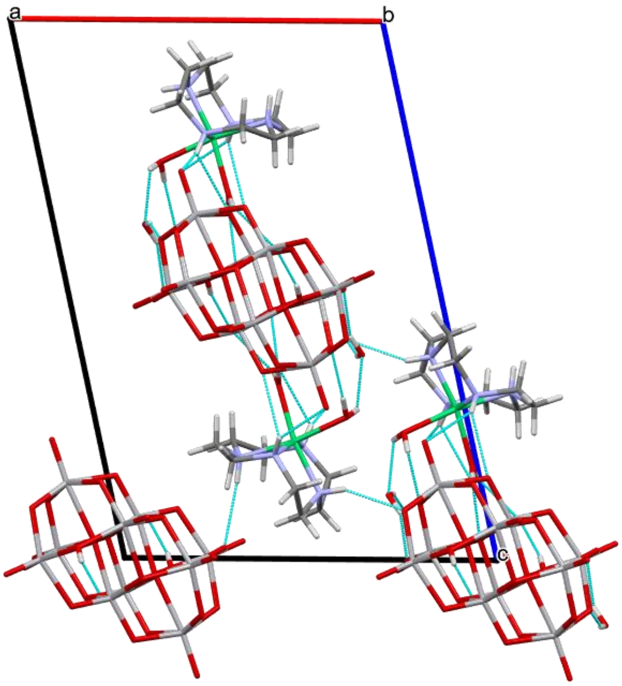

Figure S2. View along the c-axis direction of the crystal packing of $[Ni(OH_2)_2(cyclen)]_2[H_2V_{10}O_{28}]\cdot 2H_2O$ highlighting the alternating V_{10}-based chains and Ni-cyclen complexes.

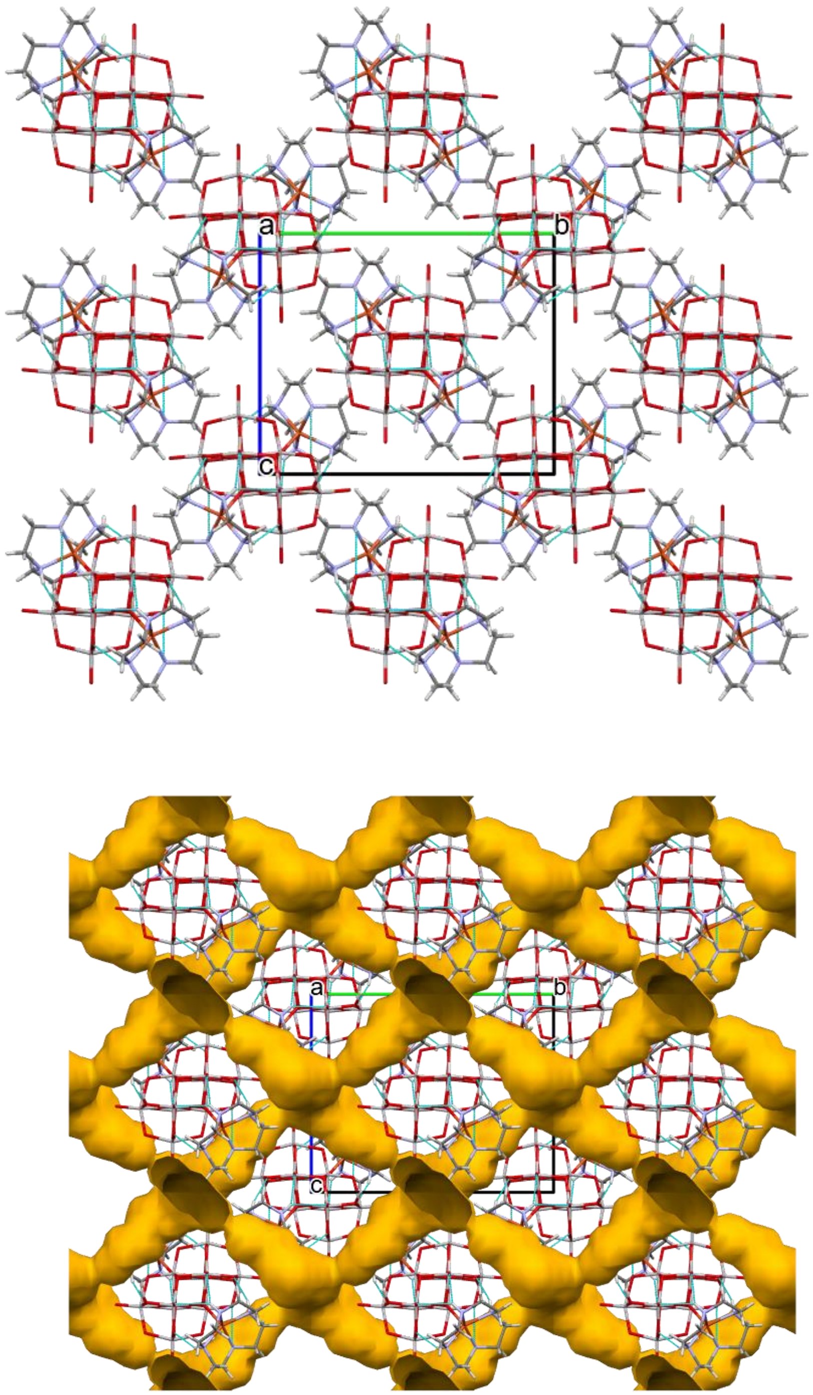

Figure S3. View along the a-axis direction of the crystal packing of $[\{Cu(cyclen)\}_2(H_2V_{10}O_{28})]\cdot 9H_2O$ (top) with highlighted the voids (bottom).

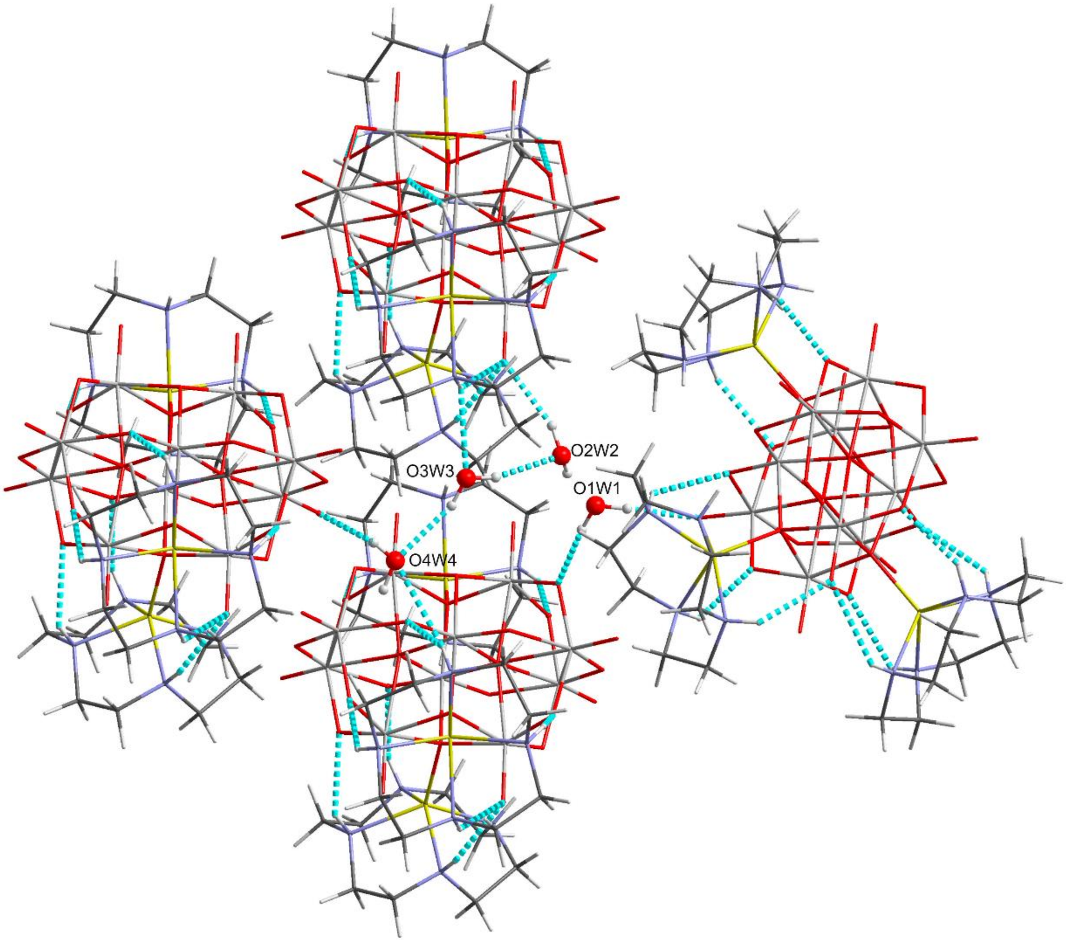

Figure S4. Intramolecular and intermolecular hydrogen bond network including the water molecules in $[\{Zn(cyclen)\}_3(V_{10}O_{28})]\cdot 4H_2O$ (**3**).

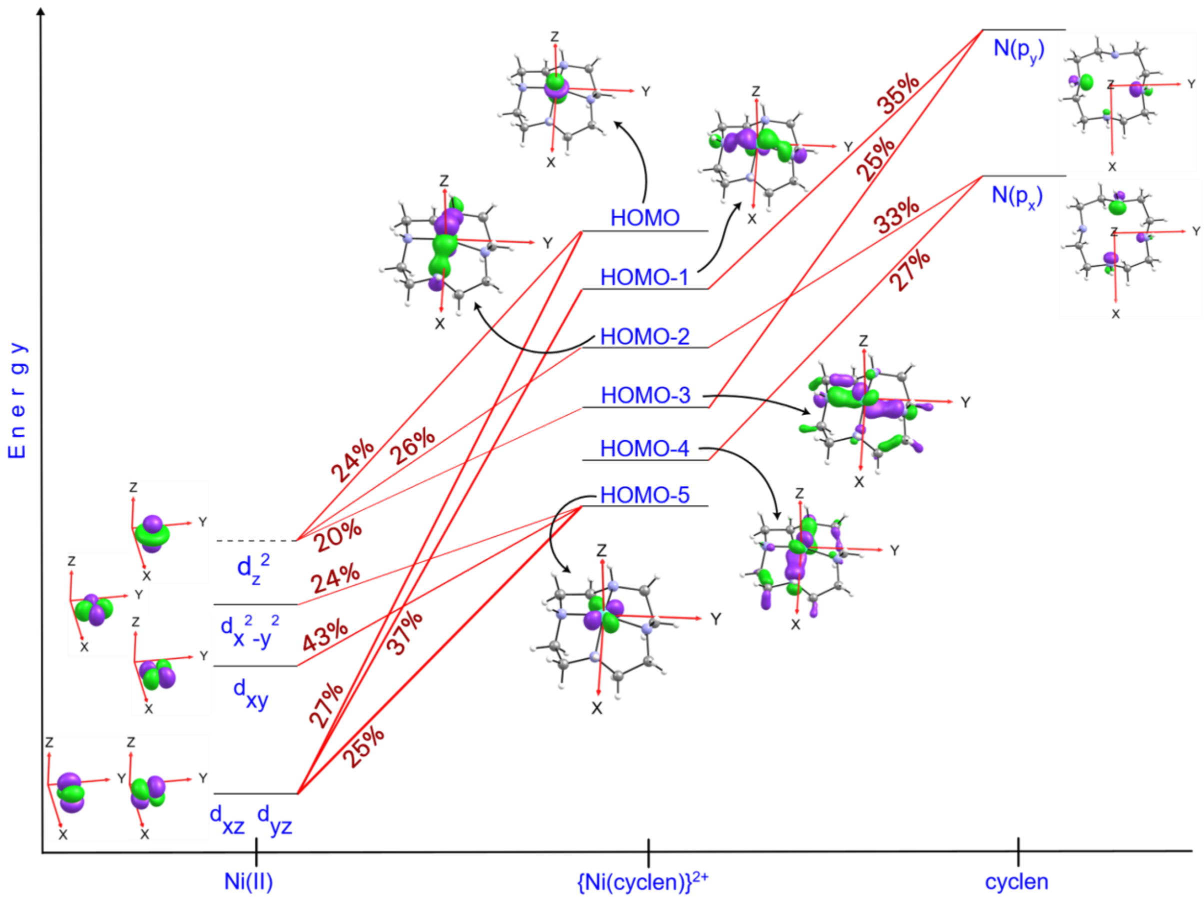

Figure S5. Charge Decomposition Analysis (CDA) of selected molecular orbitals involved in bonding between Ni(II) and the cyclen ligands in $\{Ni(cyclen)\}^{2+}$. The orbital energies were shifted for clarity, particularly for degenerated 3d orbitals. Occupied and unoccupied orbitals are represented in solid and dashed lines, respectively.

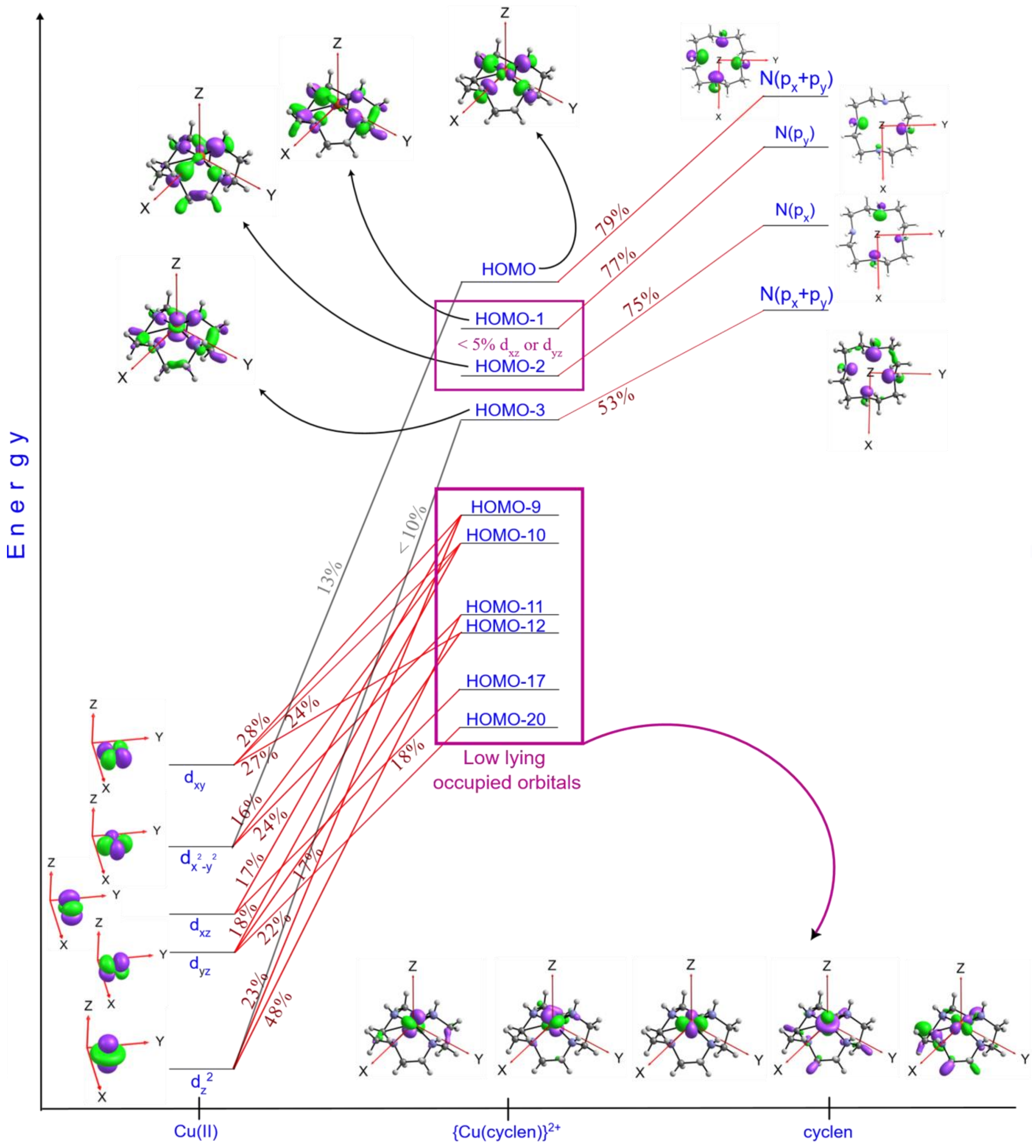

Figure S6. Charge Decomposition Analysis (CDA) of selected molecular orbitals involved in bonding between Cu(II) and the cyclen ligands in $\{Cu(cyclen)\}^{2+}$. The orbital energies were shifted for clarity, particularly for degenerated 3d orbitals.

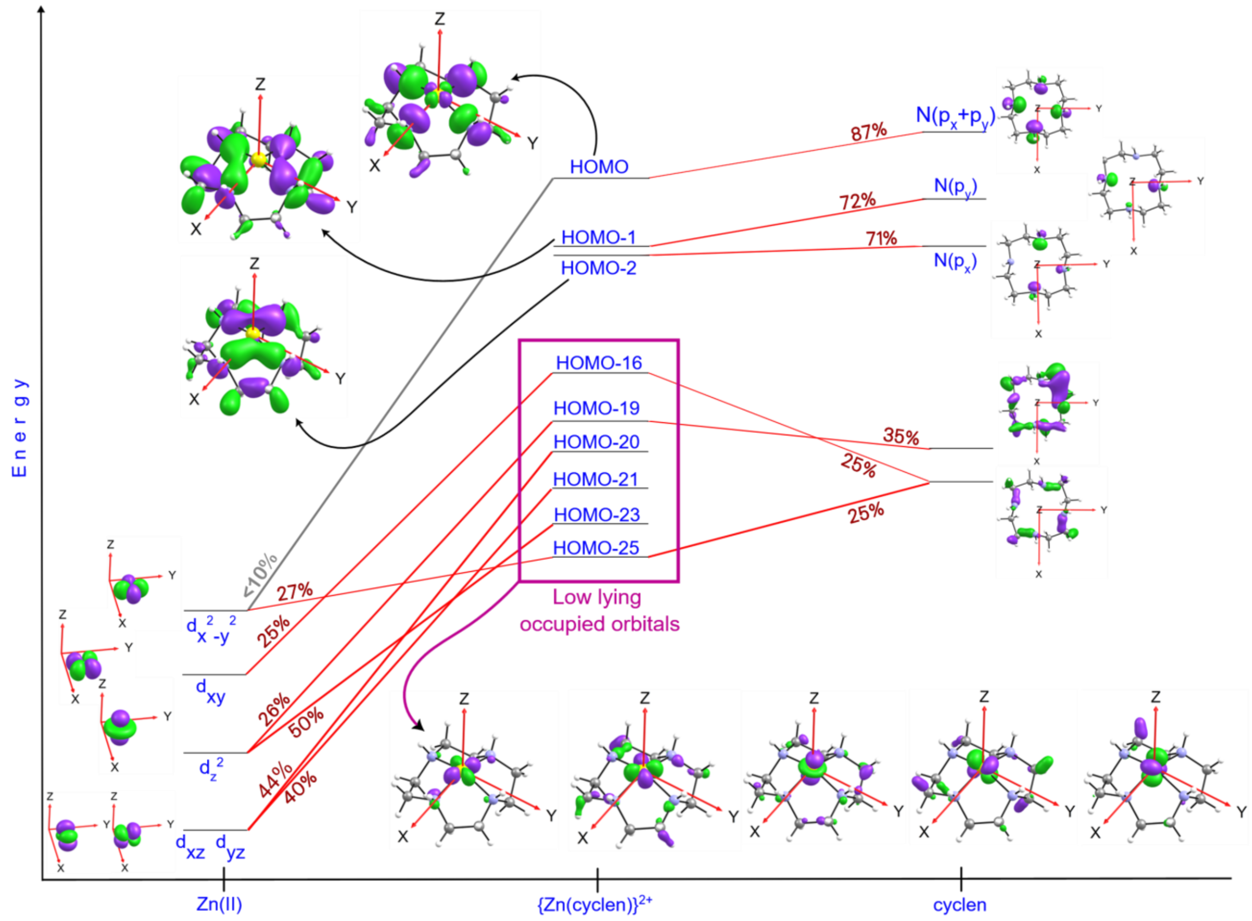

Figure S7. Charge Decomposition Analysis (CDA) of selected molecular orbitals involved in bonding between Zn(II) and the cyclen ligands in $\{Zn(cyclen)\}^{2+}$. The orbital energies were shifted for clarity, particularly for degenerated 3d orbitals.

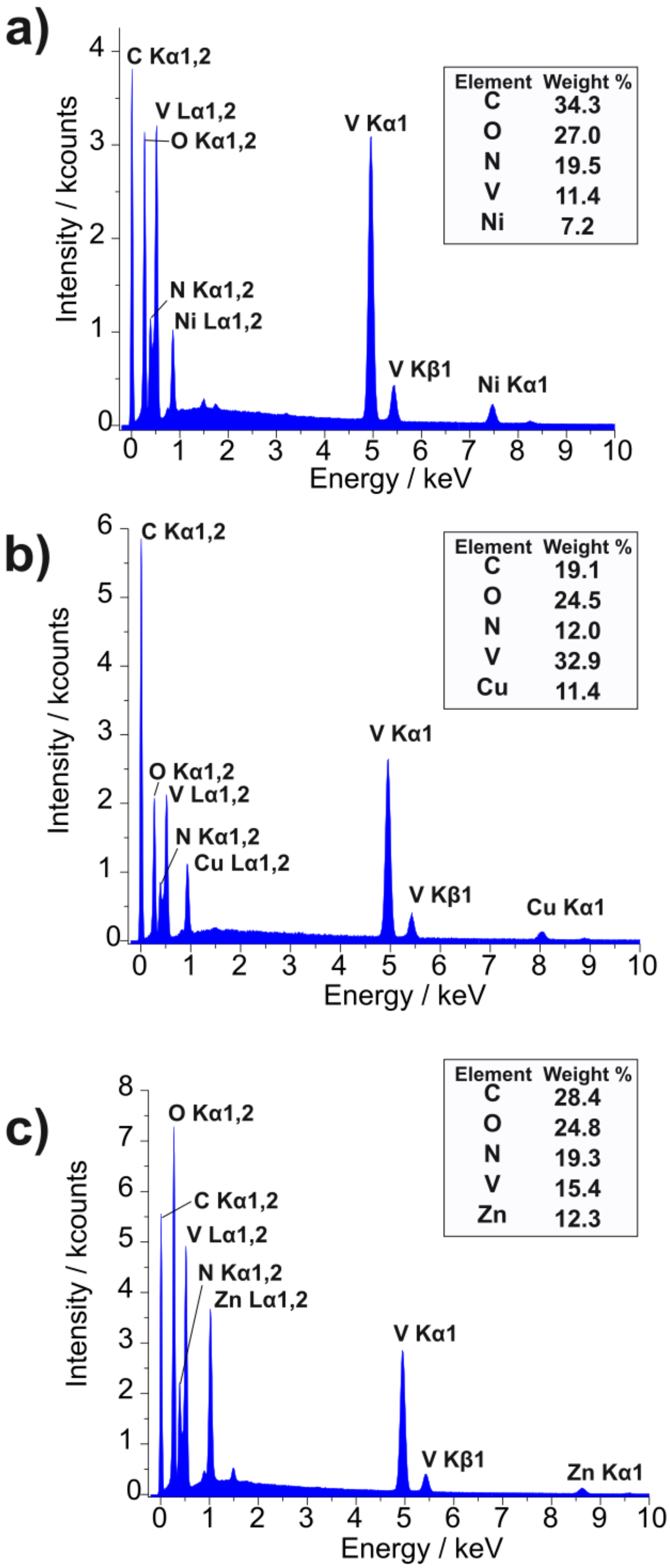

Figure S8. Energy Dispersive X-ray Spectroscopy (EDS) spectra of (a) **1,** (b) **2** and (c) **3**.

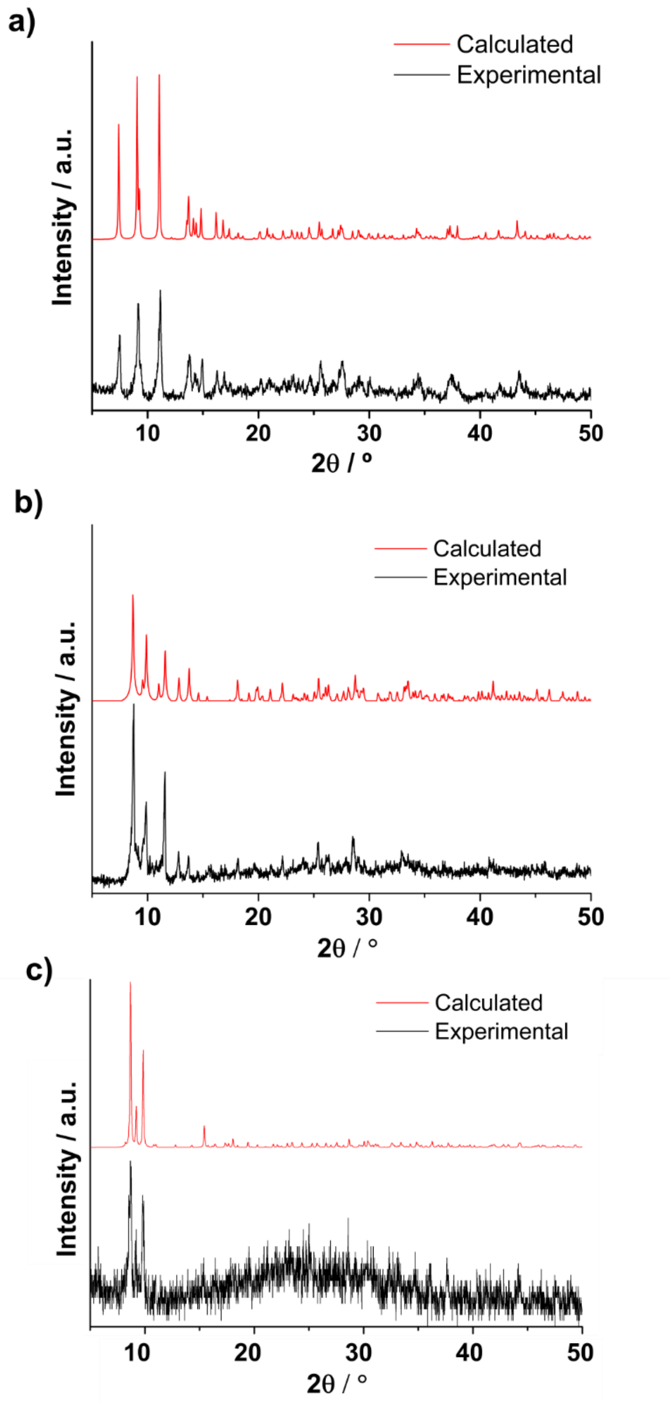

Figure S9: Comparison of calculated (red) and experimental (black) powder X-ray diffraction (PXRD) patterns for product (a) 1, (b) 2, and (c) 3. The calculated patterns were obtained from crystallographic data, while the experimental patterns confirm the phase purity and crystallinity of the synthesized compounds.

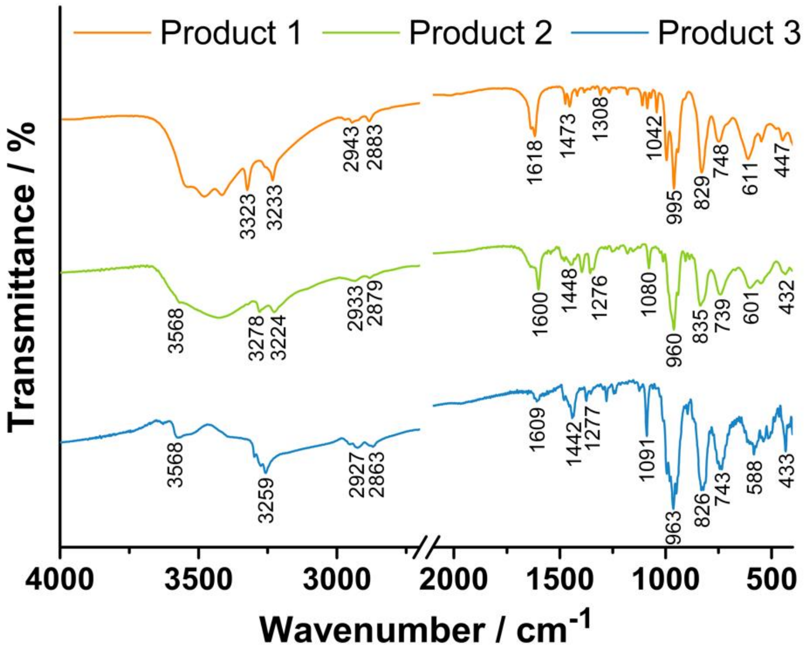

Figure S10. Comparative infrared spectra IR of **1, 2** and **3.**

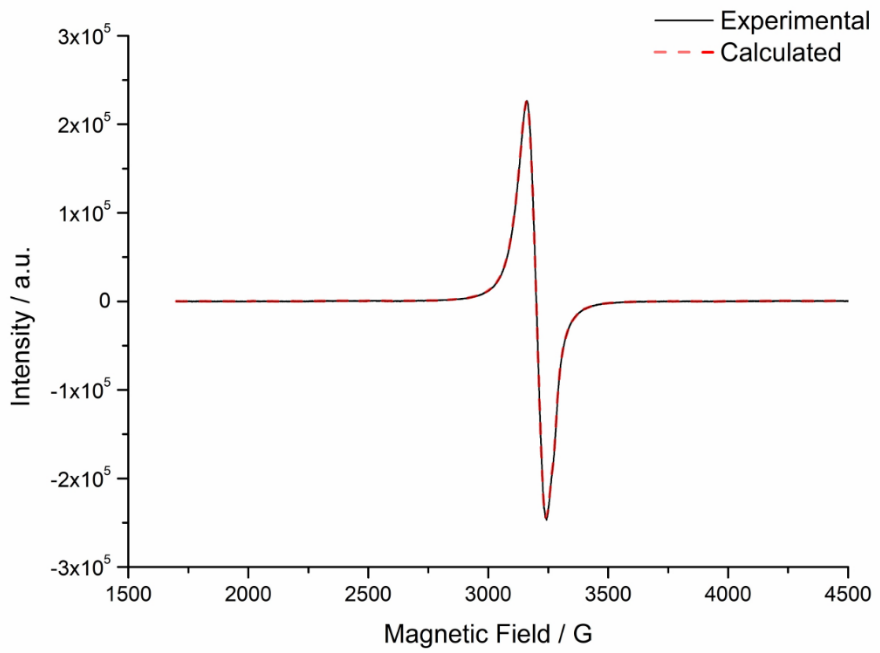

Figure S11. EPR spectrum in X–band recorded at 77 K for a pulverized sample of **2** (black) and the corresponding calculated spectrum (red).

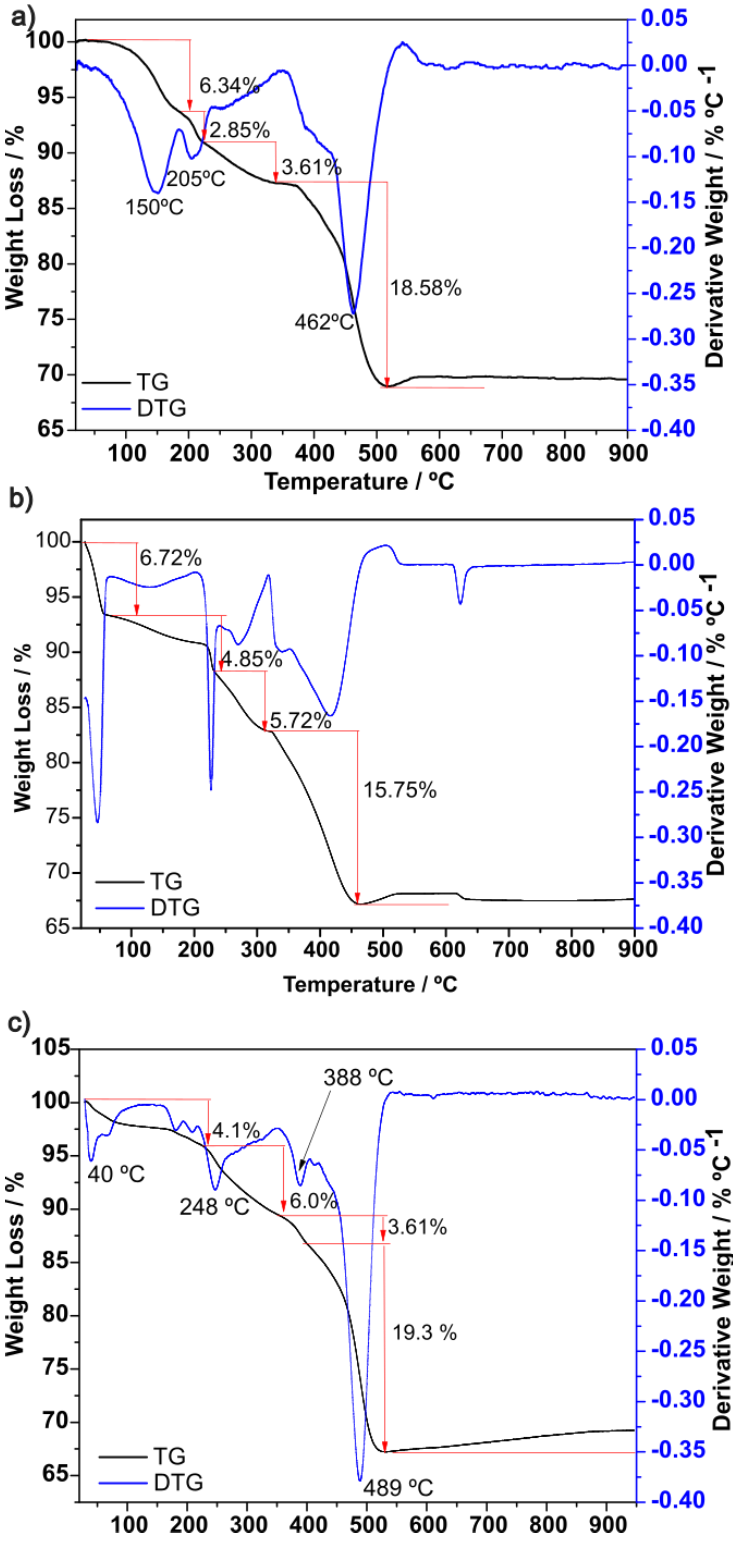

Figure S12. Thermogravimetric and DTG profiles of products **1** (a), **2** (b), and **3** (c), highlighting the mass loss steps. The thermograms were recorded in a N_2/O_2 atmosphere over a temperature range of 25–900 °C.

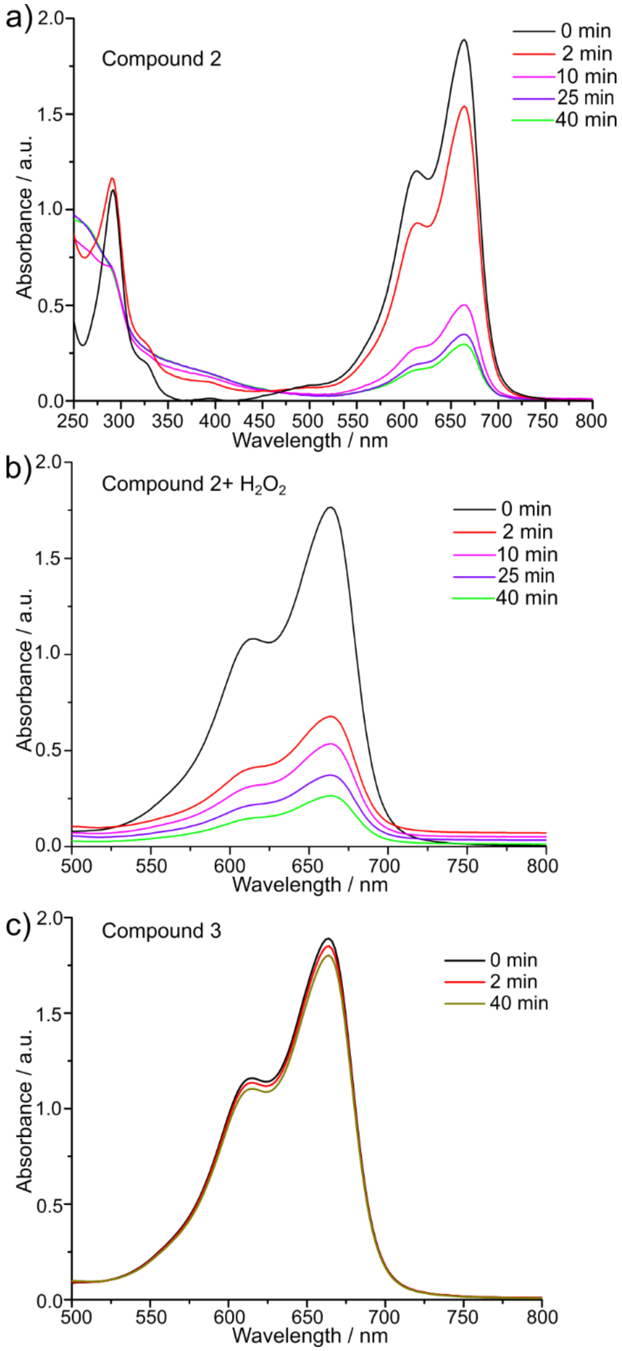

Figure S13. UV-Vis absorption spectra showing the decrease of the absorption band of methylene blue at different reaction times. (a) Full spectral range from 250 to 800 nm, in the presence of compound **2**. (b) Zoomed-in view from 500 to 800 nm, highlighting the decrease in absorbance of the band at 664 nm over time, in the presence of compound **2** and H_2O_2 and (c) absorbance spectra of methylene blue in the presence of compound **3**.

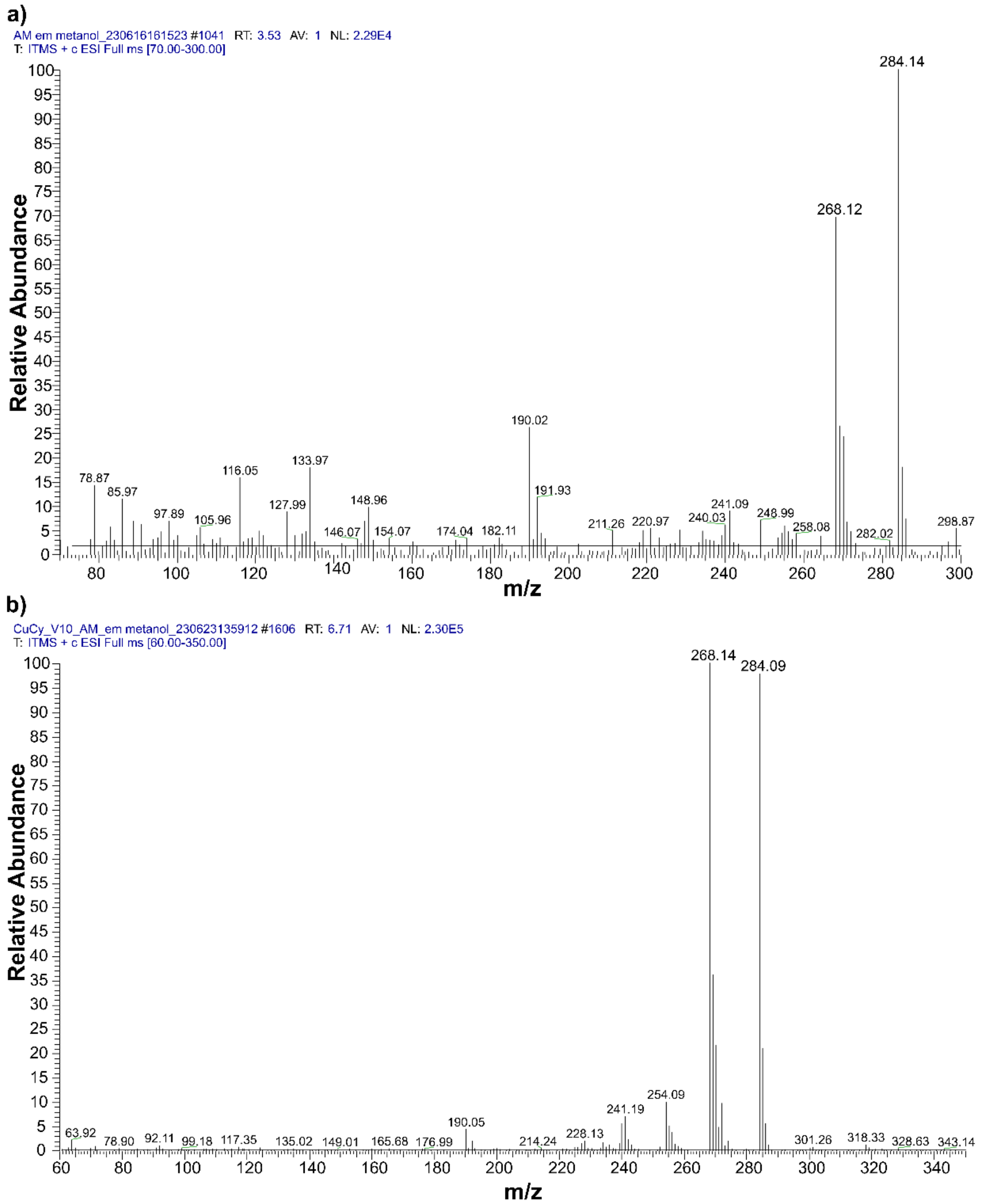

Figure S14. ESI-MS scan of MB (10 mg L^{-1}) in positive mode (a) before and (b) after the reaction with 10 mg of **2**.

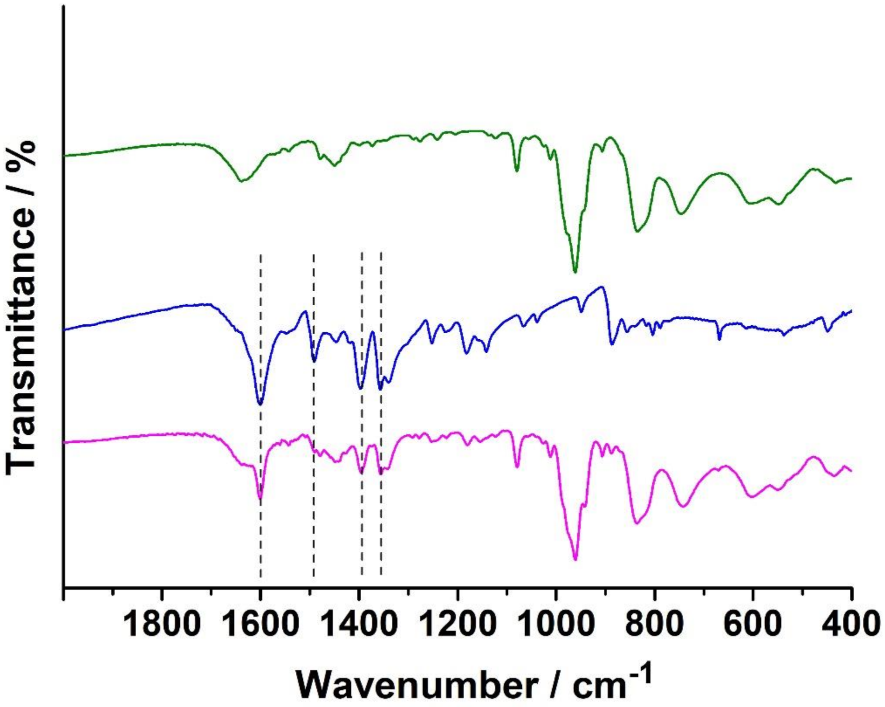

Figure S15. IR spectra registered for compound **2** (green), methylene blue (blue), and the remaining compound after interaction (pink). The dashed lines indicate characteristic vibrational bands for functional group identification and comparison.

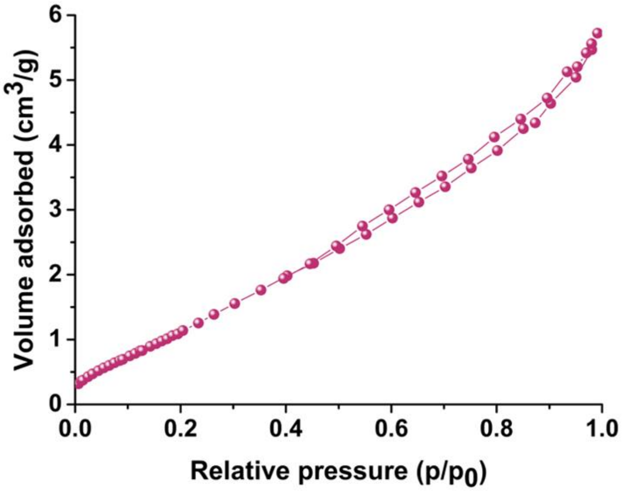

Figure S16. N_2 adsorption/desorption isotherm of **2**.

DFT Calculations: Keywords employed for all optimization calculations:

!DFT wb97x-d3 def2-tzvp opt rijcosx autoaux largeprint printbasis printmos

Table S12. {Ni(cyclen)}$^{2+}$ optimized geometry for multiplicity 1 and charge 2:

Atom label	Coordinate X / Å	Coordinate Y / Å	Coordinate Z / Å
Ni	-0.0041940	0.0002810	-0.3088990
N	-0.3048550	-1.8792120	-0.5559330
N	-1.8821740	0.3012620	-0.5645950
N	0.2982930	1.8795600	-0.5550760
N	1.8765320	-0.3015830	-0.5474460
H	-0.1568070	-2.4293760	0.2864410
C	-1.7122710	-2.0738280	-1.0121080
C	0.7537520	-2.2393980	-1.5393260
H	-2.4363160	0.1508520	0.2747410
C	-2.2383280	-0.7540130	-1.5529170
C	-2.0743690	1.7105100	-1.0167030
H	0.1433310	2.4312770	0.2852780
C	-0.7516520	2.2388780	-1.5480880
C	1.7098180	2.0733180	-0.9992780
H	2.4238700	-0.1500780	0.2961470
C	2.2405700	0.7527900	-1.5338940
C	2.0720710	-1.7110980	-0.9970430
H	-1.7812430	-2.8675240	-1.7572160
H	-2.2990250	-2.3860560	-0.1468660
H	0.7946560	-3.3183510	-1.7012380
H	0.5026900	-1.7724550	-2.4944310
H	-3.3166870	-0.7943590	-1.7188970
H	-1.7678590	-0.5001030	-2.5055040
H	-2.8636790	1.7820690	-1.7662140
H	-2.3917770	2.2938550	-0.1510580
H	-0.7914050	3.3178160	-1.7103690
H	-0.4921070	1.7717600	-2.5007990
H	1.7851980	2.8657420	-1.7451250
H	2.2890650	2.3869410	-0.1295030
H	3.3201330	0.7936540	-1.6916900
H	1.7775140	0.4977260	-2.4898310
H	2.8675620	-1.7831310	-1.7399480
H	2.3823180	-2.2941300	-0.1285880

Table S13. $\{Cu(cyclen)\}^{2+}$ optimized geometry for multiplicity 2 and charge 2:

Atom label	Coordinate X / Å	Coordinate Y / Å	Coordinate Z / Å
Cu	-0.0045570	0.0002130	-0.1230060
N	-0.3110280	-1.9501940	-0.5249570
N	-1.9521760	0.3066280	-0.5353640
N	0.3039210	1.9505640	-0.5243990
N	1.9469770	-0.3070540	-0.5174760
H	-0.1700710	-2.5774560	0.2613650
C	-1.7106390	-2.0822090	-1.0066800
C	0.7470280	-2.2078260	-1.5346700
H	-2.5839860	0.1642100	0.2470520
C	-2.2059470	-0.7482370	-1.5492480
C	-2.0819340	1.7084410	-1.0123180
H	0.1568480	2.5800080	0.2594170
C	-0.7449170	2.2073790	-1.5438950
C	1.7081250	2.0815200	-0.9937390
H	2.5717410	-0.1630140	0.2703010
C	2.2082080	0.7468520	-1.5303560
C	2.0797810	-1.7092880	-0.9920470
H	-1.7974880	-2.8620370	-1.7655410
H	-2.3225350	-2.3877300	-0.1563190
H	0.8073720	-3.2693060	-1.7830590
H	0.4761500	-1.6785450	-2.4521870
H	-3.2665280	-0.8071490	-1.8017970
H	-1.6730410	-0.4753890	-2.4640420
H	-2.8565250	1.7975600	-1.7762520
H	-2.3939090	2.3163260	-0.1614470
H	-0.8034480	3.2689430	-1.7923350
H	-0.4657750	1.6783730	-2.4590350
H	1.8016200	2.8599240	-1.7532750
H	2.3124040	2.3884150	-0.1384520
H	3.2703580	0.8067340	-1.7759190
H	1.6814650	0.4729200	-2.4484470
H	2.8604840	-1.7993430	-1.7496240
H	2.3846460	-2.3166380	-0.1382260

Table S14. $\{Zn(cyclen)\}^{2+}$ optimized geometry for multiplicity 1 and charge 2:

Atom label	Coordinate X / Å	Coordinate Y / Å	Coordinate Z / Å
Zn	-0.0055900	0.0002870	0.0637180
N	-0.3151110	-1.9968380	-0.5038520
N	-2.0011920	0.3115490	-0.5149960
N	0.3078650	1.9973300	-0.5035960
N	1.9955030	-0.3116400	-0.4966480
H	-0.1864910	-2.6907690	0.2257830
C	-1.7066930	-2.0867360	-1.0063050
C	0.7407030	-2.1894080	-1.5254630
H	-2.6998870	0.1808720	0.2096280
C	-2.1866130	-0.7407540	-1.5410960
C	-2.0879430	1.7054430	-1.0117410
H	0.1736490	2.6928720	0.2237410
C	-0.7386650	2.1887930	-1.5348450
C	1.7041190	2.0861760	-0.9937900
H	2.6878250	-0.1798200	0.2338240
C	2.1891500	0.7395290	-1.5222240
C	2.0856100	-1.7060950	-0.9912730
H	-1.8002050	-2.8487400	-1.7826720
H	-2.3370210	-2.3977740	-0.1711450
H	0.8165570	-3.2369270	-1.8230810
H	0.4548790	-1.6267590	-2.4187000
H	-3.2320700	-0.8162410	-1.8460490
H	-1.6182670	-0.4528080	-2.4300070
H	-2.8443450	1.8022680	-1.7931880
H	-2.4054630	2.3314900	-0.1758090
H	-0.8122240	3.2361650	-1.8335360
H	-0.4450690	1.6258310	-2.4253050
H	1.8041830	2.8467230	-1.7707450
H	2.3269100	2.3989020	-0.1535970
H	3.2367080	0.8158060	-1.8196290
H	1.6272780	0.4500090	-2.4148350
H	2.8484180	-1.8037160	-1.7663640
H	2.3961130	-2.3314250	-0.1521780

References

1. Huang, X.; Qi, Y.; Gu, Y.; Gong, S.; Shen, G.; Li, Q.; Li, J., Imidazole-directed fabrication of three polyoxovanadate-based copper frameworks as efficient catalysts for constructing C–N bonds. *Dalton Trans.* **2020,** *49* (31), 10970-10976.
2. Ou, G.-C.; Jiang, L.; Feng, X.-L.; Lu, T.-B., Vanadium polyoxoanion-bridged macrocyclic metal complexes: from one-dimensional to three-dimensional structures. *Dalton Trans.* **2009,** (1), 71-76.
3. Klištincová, L.; Rakovský, E.; Schwendt, P., Decavanadate ion as bridging ligand. Synthesis and crystal structure of $(NH_4)_2[Cu_2(NH_3CH_2CH_2COO)_4(V_{10}O_{28})]\cdot10H_2O$. *Inorg. Chem. Commun.* **2008,** *11* (10), 1140-1142.
4. Martín-Caballero, J.; San José Wéry, A.; Reinoso, S.; Artetxe, B.; San Felices, L.; El Bakkali, B.; Trautwein, G.; Alcañiz-Monge, J.; Vilas, J. L.; Gutiérrez-Zorrilla, J. M., A Robust Open Framework Formed by Decavanadate Clusters and Copper(II) Complexes of Macrocyclic Polyamines: Permanent Microporosity and Catalytic Oxidation of Cycloalkanes. *Inorg. Chem.* **2016,** *55* (10), 4970-4979.
5. Thomas, J.; Agarwal, M.; Ramanan, A.; Chernova, N.; Whittingham, M. S., Copper pyrazole directed crystallization of decavanadates: synthesis and characterization of $\{Cu(pz)\}_4[\{Cu(pz)_3\}_2V_{10}O_{28}]$ and $(Hpz)_2[\{Cu(pz)_4\}_2V_{10}O_{28}]\cdot2H_2O$. *CrystEngComm* **2009,** *11* (4), 625-631.
6. Zhang, Q.; Ondus, J.; Mills, J.; Bahadori, A.; Smith, J.; Jordan, T.; Xu, H.; Hwu, S.-J., Bench-top electrochemical crystal growth of POMOFs: In-situ synthesis of new organic-inorganic hybrids containing $[V_{10}O_{28}]^{(6+n)-}$ polyoxovanadate. *J. Solid State Chem.* **2020,** *287*, 121368.
7. Pavliuk, M. V.; Makhankova, V. G.; Khavryuchenko, O. V.; Kokozay, V. N.; Omelchenko, I. V.; Shishkin, O. V.; Jezierska, J., Decavanadates decorated with $[Cu(en)_2]^{2+}$: Convenient synthetic route, crystal structures and analysis of vibrational spectra. *Polyhedron* **2014,** *81*, 597-606.
8. Li, T.; Lü, J.; Gao, S.; Li, F.; Cao, R., Inorganic–Organic Hybrid with 3D Supramolecular Channel Assembled through C–H···O Interactions Based on the Decavanadate. *Chem. Lett.* **2007,** *36* (3), 356-357.
9. Bartošová, L.; Padělková, Z.; Rakovský, E.; Schwendt, P., Synthesis and crystal structure of two copper(II) complexes with coordinated decavanadate ion. *Polyhedron* **2012,** *31* (1), 565-569.
10. Ma, H.; Meng, X.; Sha, J.; Pang, H.; Wu, L., Synthesis, crystal structure and properties of a new bi-dentate decavanadate $[Cu(en)_2H_2O]_2[H_2V_{10}O_{28}]\cdot12H_2O$. *Solid State Sci.* **2011,** *13* (5), 850-854.
11. An, L.; Liu, X.; Zhou, J.; Hu, F.; Zhu, L., A New 1D Polyoxovanadate $[Cu(en)_2V_{10}O_{28}][Cu(en)_2(H_2O)]_2$ $2H_3BO_3$ $2H_2O$ Containing the Rarely Non-condensed Boric acid. *Z. Naturforsch. B* **2012,** *67* (9), 860-864.
12. Li, C.; Zhong, D.; Huang, X.; Shen, G.; Li, Q.; Du, J.; Li, Q.; Wang, S.; Li, J.; Dou, J., Two organic–inorganic hybrid polyoxovanadates as reusable catalysts for Knoevenagel condensation. *New J. Chem.* **2019,** *43* (15), 5813-5819.
13. Dou, M.-Y.; Zhong, D.-D.; Huang, X.; Yang, G.-Y., Imidazole-induced self-assembly of polyoxovanadate cluster organic framework for efficient Knoevenagel condensation under mild conditions. *CrystEngComm* **2020,** *22*.

14. Huang, X.; Gu, X.; Zhang, H.; Shen, G.; Gong, S.; Yang, B.; Wang, Y.; Chen, Y., Decavanadate-based clusters as bifunctional catalysts for efficient treatment of carbon dioxide and simulant sulfur mustard. *J. CO_2 Util.* **2021,** *45*, 101419.
15. Pang, H.; Meng, X.; Ma, H.; Liu, B.; Li, S., An Unusual Chain Structure of Decavanadates $V_{10}O_{28}$ Linked by $Zn(en)_2$ Units. *Z. Naturforsch. B* **2012,** *67* (9), 855-859.
16. Wang, M.; Sun, W.; Pang, H.; Ma, H.; Yu, J.; Zhang, Z.; Niu, Y.; Yin, M., Tuning the microstructures of decavanadate-based supramolecular hybrids via regularly changing the spacers of bis(triazole) ligands. *J. Solid State Chem.* **2016,** *235*, 175-182.
17. Wang, L.; Sun, X.-p.; Liu, M.-l.; Gao, Y.-q.; Gu, W.; Liu, X., Syntheses, Structures and Properties of Three Heteonuclear Complexes Containing $[V_{10}O_{28}]^{6-}$ Units. *J. Cluster Sci.* **2008,** *19* (3), 531-542.
18. Xu, W.; Jiang, F.; Zhou, Y.; Xiong, K.; Chen, L.; Yang, M.; Feng, R.; Hong, M., Three novel organic–inorganic complexes based on decavanadate $[V_{10}O_{28}]^{6-}$ units: special water layers, open 3D frameworks and yellow/blue luminescences. *Dalton Trans.* **2012,** *41* (25), 7737-7745.
19. Amanchi, S. R.; Das, S. K., A Versatile Polyoxovanadate in Diverse Cation Matrices: A Supramolecular Perspective. *Front. Chem.* **2018,** *6*.
20. Somasundaram, J. D.; Ebrahimi, A.; Nandan, S. P.; Cherevan, A.; Eder, D.; Šupolíková, M.; Nováková, E.; Gyepes, R.; Krivosudský, L., Functionalization of decavanadate anion by coordination to cobalt(II): Binding to proteins, cytotoxicity, and water oxidation catalysis. *J. Inorg. Biochem.* **2023,** *239*, 112067.
21. Krivosudský, L.; Roller, A.; Rompel, A., Tuning the interactions of decavanadate with thaumatin, lysozyme, proteinase K and human serum proteins by its coordination to a pentaaquacobalt(ii) complex cation. *New J. Chem.* **2019,** *43* (45), 17863-17871.
22. Huang, X.; Cui, Y.; Zhou, J.; Zhang, Y.; Shen, G.; Yao, Q.; Li, J.; Xue, Z.; Yang, G., Self-assembly of three Ag-polyoxovanadates frameworks for their efficient construction of CN bond and detoxification of simulant sulfur mustard. *Chin. Chem. Lett.* **2022,** *33* (5), 2605-2610.
23. Qi, Y.; Wang, E.; Li, J.; Li, Y., Two organic–inorganic poly(pseudo-rotaxane)-like composite solids constructed from polyoxovanadates and silver organonitrogen polymers. *J. Solid State Chem.* **2009,** *182* (10), 2640-2645.
24. McGlone, T.; Thiel, J.; Streb, C.; Long, D.-L.; Cronin, L., An unprecedented silver-decavanadate dimer investigated using ion-mobility mass spectrometry. *Chem. Commun.* **2012,** *48* (3), 359-361.
25. Streb, C.; Tsunashima, R.; MacLaren, D. A.; McGlone, T.; Akutagawa, T.; Nakamura, T.; Scandurra, A.; Pignataro, B.; Gadegaard, N.; Cronin, L., Supramolecular Silver Polyoxometalate Architectures Direct the Growth of Composite Semiconducting Nanostructures. *Angew. Chem. Int. Ed.* **2009,** *48* (35), 6490-6493.
26. Li, J.-K.; Wei, C.-P.; Wang, Y.-Y.; Zhang, M.; Lv, X.-R.; Hu, C.-W., Conversion of V_6 to V_{10} cluster: Decavanadate-based Mn-polyoxovanadate as robust heterogeneous catalyst for sulfoxidation of sulfides. *Inorg. Chem. Commun.* **2018,** *87*, 5-7.
27. Franco, M. P.; Rüdiger, A. L.; Soares, J. F.; Nunes, G. G.; Hughes, D. L., Crystal structures of two deca-vanadates(V) with penta-aqua-manganese(II) pendant groups: $(NMe_4)_2[V_{10}O_{28}\{Mn(H_2O)_5\}_2]\cdot 5H_2O$ and $[NH_3C(CH_2OH)_3]_2[V_{10}O_{28}\{Mn(H_2O)_5\}_2]\cdot 2H_2O$. *Acta Crystallogr. Sect. E: Cryst. Commun.* **2015,** *71* (Pt 2), 146-50.
28. Avila, P. F.; Ripplinger, T. J.; Kemper, D. J.; Domine, J. L.; Jordan, C. D., Features of Vibrational and Electronic Structures of Decavanadate Revealed by Resonance Raman Spectroscopy and Density Functional Theory. *J. Phys. Chem. Lett.* **2019,** *10* (20), 6032-6037.
